# Distinct roles of Arabidopsis ORC1 proteins in DNA replication and heterochromatic H3K27me1 deposition

**DOI:** 10.1038/s41467-023-37024-8

**Published:** 2023-03-07

**Authors:** Zaida Vergara, María S. Gomez, Bénédicte Desvoyes, Joana Sequeira-Mendes, Kinda Masoud, Celina Costas, Sandra Noir, Elena Caro, Victoria Mora-Gil, Pascal Genschik, Crisanto Gutierrez

**Affiliations:** 1 Centro de Biología Molecular Severo Ochoa, CSIC-UAM, Nicolás Cabrera 1, Cantoblanco, 28049 Madrid, Spain; 2 Institut de Biologie Moléculaire des Plantes, CNRS, Université de Strasbourg, 12 rue Général Zimmer, 67084 Strasbourg cedex, France; 3 Present Address: Biotechnology and Health research group, ANFACO-CECOPESCA, 36310 Vigo, Spain; 4 Present Address: Centre for Plant Biotechnology and Genomics, Departamento de Biotecnología-Biología Vegetal, Universidad Politécnica de Madrid (UPM) - Instituto Nacional de Investigación y Tecnología Agraria y Alimentaria (INIA), 28223 Pozuelo de Alarcón, Spain

**Keywords:** Plant cell cycle, Plant molecular biology

## Abstract

Most cellular proteins involved in genome replication are conserved in all eukaryotic lineages including yeast, plants and animals. However, the mechanisms controlling their availability during the cell cycle are less well defined. Here we show that the Arabidopsis genome encodes for two ORC1 proteins highly similar in amino acid sequence and that have partially overlapping expression domains but with distinct functions. The ancestral *ORC1b* gene, present before the partial duplication of the Arabidopsis genome, has retained the canonical function in DNA replication. ORC1b is expressed in both proliferating and endoreplicating cells, accumulates during G1 and is rapidly degraded upon S-phase entry through the ubiquitin-proteasome pathway. In contrast, the duplicated *ORC1a* gene has acquired a specialized function in heterochromatin biology. ORC1a is required for efficient deposition of the heterochromatic H3K27me1 mark by the ATXR5/6 histone methyltransferases. The distinct roles of the two ORC1 proteins may be a feature common to other organisms with duplicated *ORC1* genes and a major difference with animal cells.

## Abstract (web summary)

The dynamics of DNA replication factors is crucial for cell cycle progression. Arabidopsis encode two origin recognition complex 1 proteins, ORC1a and ORC1b, with distinct roles in DNA replication and deposition of heterochromatic H3K27me1, respectively.

## Introduction

The cell cycle is a highly regulated and conserved cellular process aiming at producing two daughter cells. After cell division, the G1 period is characterized by different events including a transcriptional wave required to produce factors needed for DNA and chromatin duplication^1–3^. DNA replication takes place during the S-phase once DNA replication origins (ORIs) are activated. Chromatin dynamics plays a primary role during the cell cycle where chromatin marks are relevant for both eu- and heterochromatin organization.

ORIs are the genomic sites where DNA replication initiates. All potential ORIs are marked in the genome by the presence of pre-replication complexes (pre-RCs) that are loaded into chromatin early in G1. The origin recognition complex (ORC), a heterohexameric complex (ORC1-6), is the first to get access to chromatin. Its ATPase activity provides the necessary energy for the subsequent loading of the other pre-RC components. Briefly, once the ORC and CDC6 are loaded onto chromatin^4^, CDT1 binds to the MCM2-7 hexamer to complete pre-RC assembly^5–8^.

Pre-RC components are highly conserved in all eukaryotic lineages, including yeasts, plants and animals^9–11^. However, the regulatory mechanisms controlling the availability of these proteins have varied enormously during evolution. For instance, in plants all ORC subunits, except ORC5, are regulated by the RBR-E2F pathway^12–14^ whereas in animal cells, only the large subunit ORC1 is an E2F target^15^. ORC in yeast remains bound to chromatin during the entire cell cycle whereas in animals, one or more subunits are evicted from the pre-RC complex after origin activation in S-phase and either exported out of the nucleus or targeted for proteasome degradation^16–19^. In spite of early studies in plants^12,13,20^, our knowledge of the dynamics of pre-RC components in relation to cell cycle progression lags behind. In addition, ORC1, CDC6 and CDT1 proteins are encoded by duplicated genes in *Arabidopsis thaliana*^21^, posing the challenge of understanding whether they are functionally redundant or play unique roles. In addition, compared to other systems, the postembryonic development of plants offers a unique opportunity to study pre-RC dynamics in different cell types during organogenesis.

Here we have investigated the Arabidopsis ORC1 proteins, their cellular localization and the mutant phenotypes to define their functional roles. We found that the two proteins have distinct expression domains and functions. ORC1b is cell cycle regulated, starts to accumulate at low levels in mid-late G2, is maintained during mitosis and fully loaded onto chromatin during G1. Then it rapidly disappears upon S-phase entry by being targeted for proteasome degradation. ORC1b is involved in DNA replication as part of the pre-RCs that mark potential ORIs and is needed for efficient entry in S-phase. On the contrary, ORC1a, which appears to be restricted to endoreplicating cells, has a different function in heterochromatin maintenance facilitating the deposition of H3K27me1 by the ATXR5/6 methyltransferases. Together, our results indicate that in contrast to metazoans, *Arabidopsis* has acquired two *ORC1* genes during evolution with two distinct functions in genome dynamics and stability.

## Results

### Expression domains of ORC1

**Fig. 1:**
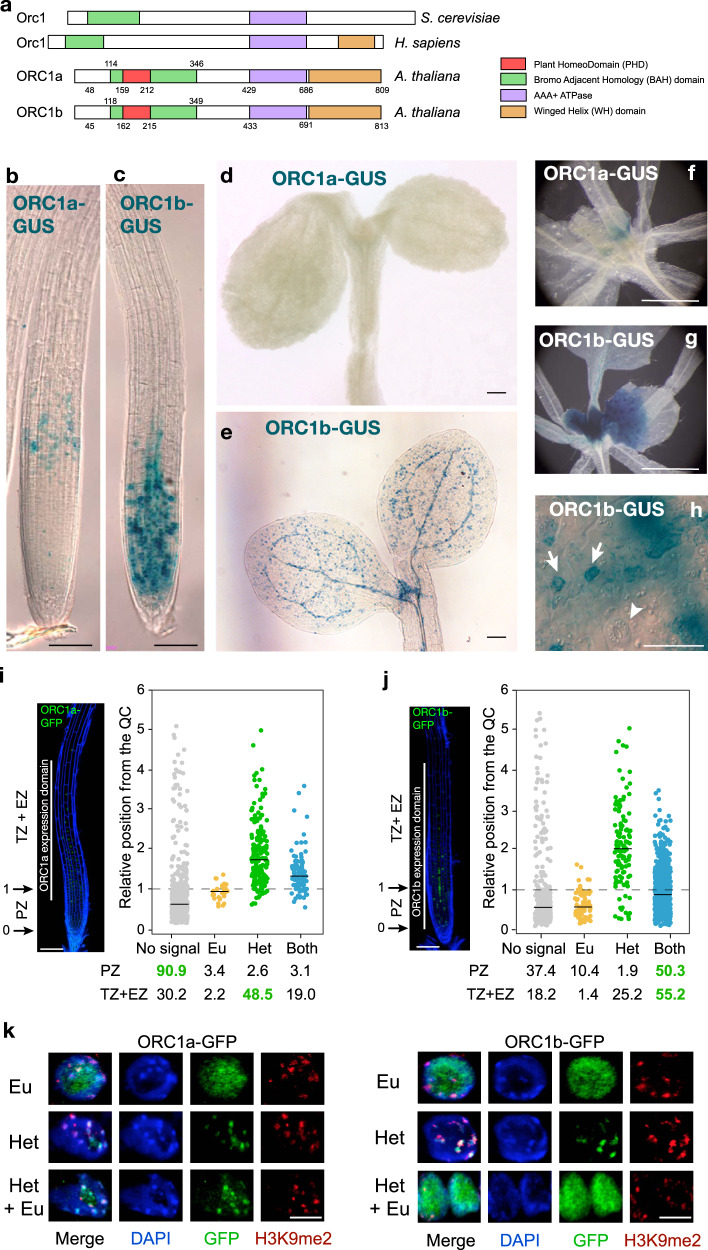
Expression domains and nuclear patterns of Arabidopsis ORC1a and ORC1b proteins. **a** Domain organization of *S. cerevisiae* Orc1, human Orc1 and *A. thaliana* ORC1a and ORC1b. Detection of ORC1a-GUS (**b**, **d**, **f**) and ORC1b-GUS (**c**, **e**, **g**, **h**) in the root apex of 7 day-old seedlings (**b**, **c**), aerial part of 4 day-old seedlings (**d**, **e**), 12 day-old seedlings (**f**, **g**) and meristemoids (arrows) and mature guard cells (arrowhead) of the stomatal lineage (**h**). Images are representative of at least 6 independent lines, which showed similar expression patterns. Scale bars = 100 µm, except in panels **f** and **g**, where it corresponds to 1 mm, and in panel **h**, where it corresponds to 50 µm. Nuclear expression patterns of ORC1a-GFP (**i**) and ORC1b-GFP (**j**) in the epidermis of the primary root apex of 7 day-old seedlings. The percentage of different nuclear patterns are shown in the X-axis and were quantified according to the position along the root apex, as indicated in the Y-axis. Position 0 corresponds to the QC and position 1 to the root apical meristem (RAM) boundary. The solid black lines mark the median values of each distribution (for ORC1a-GFP, *n* = 972 nuclei recorded from 9 roots and for ORC1b-GFP, *n* = 1111 nuclei from 9 roots; Supplementary Data 1). A root showing the overall GFP labeling pattern with each ORC1 protein is included on the left side of each panel. Cell walls were stained with propidium iodide (blue). No signal (grey); Eu, euchromatin (orange); Het, heterochromatin (green); Both, euchromatin and heterochromatin (light blue) labeling; the most common patterns present in the PZ or TZ + EZ are highlighted in green. PZ Proliferation zone, TZ transition zone, EZ elongation zone. Scale bars = 100 µm. **k** Examples of nuclei after immunodetection of ORC1-GFP proteins, as indicated, and the typical heterochromatic mark H3K9me2 (*n* = 2 immunodetection experiments, *n* = 6 roots/genotype, showing similar results). Scale bars = 10 µm.

The Arabidopsis genome contains two *ORC1* genes, *ORC1a* and *ORC1b*, that arose from one partial genome duplication^21^. These two genes encode for two different proteins that, nonetheless are 87% identical in amino acid sequence, and differ mainly in their 50-60 N-terminal amino acids^11,14^. Both proteins contain the major domains shared with animal and yeast Orc1 proteins, including the BAH and AAA + domains (Fig. 1a). In addition, plant ORC1 proteins share with human Orc1 a winged-helix domain near the C-terminus^11^ and possess a PHD, embedded within the BAH domain^22^. This similarity in domain organization could suggest that they play similar functions. However, in a previous study we found that *ORC1a* and *ORC1b* genes exhibit a tissue specific expression. By using a transcriptional GUS reporter line we found that *ORC1b* expression colocalizes with proliferating cells whereas in situ hybridization experiments suggested that *ORC1a* might be confined to endoreplicating cells, e.g. in the hypocotyl hook^14^. To define more precisely the expression domain of the ORC1 proteins in the Arabidopsis organs, we generated translational reporter lines expressing ORC1a and ORC1b fused to GUS under their endogenous promoters. In the root apex, ORC1a-GUS was expressed at low levels in the endoreplicating cells located in the transition zone and was not detectable in other organs, except at the base of cotyledons and leaves (Fig. 1b, d, f). On the contrary, ORC1b-GUS was detected in the root (RAM) and shoot apical meristems (SAM) (Fig. 1c, e), in cells of the stomatal lineage at early stages of development (Fig. 1e, h) but not in mature guard cells (Fig. 1h), and in the basal moiety of younger leaves (Fig. 1g), tissues characterized by the presence of actively proliferating cells.

To determine ORC1 localization at the cellular and subcellular levels we used translational reporter lines expressing ORC1-GFP fusion proteins expressed under the control of their endogenous promoters. Analysis of these lines by confocal microscopy confirmed that the two ORC1 proteins localized to the nucleus and exhibited distinct expression domains in the root. ORC1b was detected in proliferating cells in the RAM as well as in endoreplicating cells in the transition zone whereas ORC1a was specifically detected in the endoreplicating cells prior to exit to differentiation (Fig. 1i, j).

A closer inspection of the GFP-labeled nuclei revealed the occurrence of different nuclear patterns for the two ORC1 proteins. Globally, a very large percentage of cells in the root were devoid of ORC1a-GFP (90.9% in the proliferation zone (PZ) and 30.2% in the transition and elongation zones (TZ + EZ; Fig. 1i and Supplementary Data 1). Outside the RAM, a majority of ORC1a-GFP positive nuclei showed a clear punctate pattern (48.5%), likely chromocenters (Fig. 1i; see also below; Supplementary Data 1). The root-specific pattern of ORC1b-GFP was completely different compared with ORC1a-GFP since most nuclei contained ORC1b-GFP (50.3% and 55.2% in PZ and TZ + EZ, respectively; Fig. 1j; Supplementary Data 1). At the subnuclear level, ORC1b-GFP can appear with a punctate pattern, homogenously distributed in the nuclei or both (Fig. 1j; see also below; Supplementary Data 1). By immunodetection of ORC1-GFP proteins and the typical heterochromatic mark H3K9me2 together with DAPI staining, we confirmed that ORC1a-GFP colocalizes with chromocenters (Fig. 1k). These results reveal that the two ORC1 proteins are nuclear and have different expression domains: while ORC1a is preferentially localized in the heterochromatic chromocenters of endoreplicating cells, ORC1b is expressed in both proliferating and endoreplicating cells. These features indicate that ORC1a and ORC1b possess different dynamics during the cell cycle and we hypothesized that they play distinct roles.

### ORC1b starts to accumulate in G2 and reaches maximum levels in G1

**Fig. 2:**
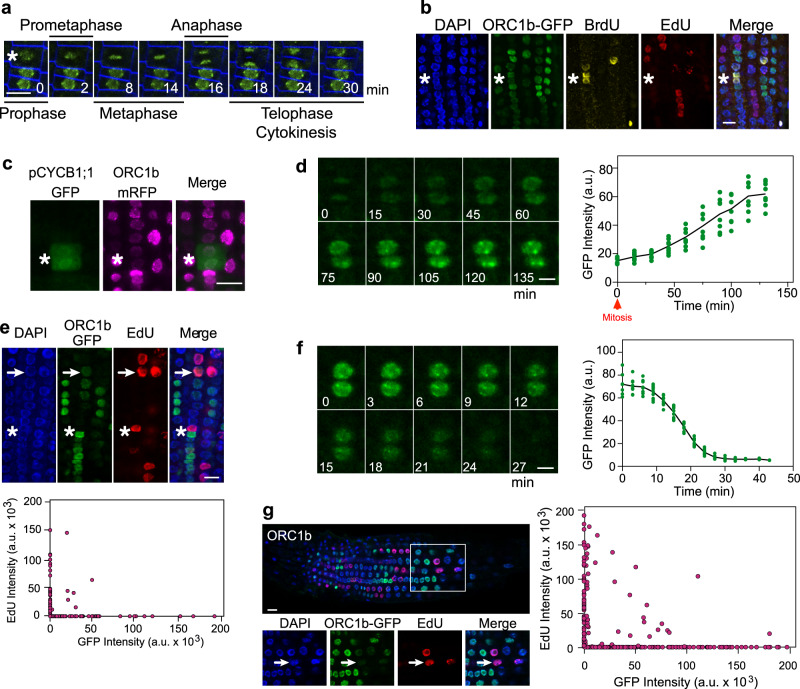
ORC1b dynamics during the cell cycle. **a** Representative live imaging of ORC1b-GFP roots (*n* = 6 movies) during mitosis. The asterisk marks a cell entering mitosis. ORC1b-GFP is shown at different time points, as indicated. The cellular plasma membrane was stained with FM4-64 (blue). **b** Identification of cells in G2 by immunofluorescent detection (*n* = 5 experiments). Roots were labeled for 15 min with BrdU (yellow), chased for 2 h with thymidine and then labeled with EdU (red) for 15 min. The GFP signal (green) was immunodetected and all the nuclei identified by DAPI staining (blue). Asterisk marks two nuclei in G2 (BrdU + , EdU–) containing ORC1b-GFP. **c** Representative image (*n* = 9) of roots expressing ORC1b-mRFP (magenta) in G2 cells expressing CYCB1;1-GFP (green), as illustrated in the cell labeled with an asterisk. **d** Representative live imaging of ORC1b-GFP roots (*n* = 6 movies) during G1. Two cells that just finished cytokinesis are shown at different time points, as indicated (left panel). The GFP intensity during ORC1b-GFP loading was quantified (right panel; a.u.: arbitrary units; Supplementary Data 1). **e** *Upper panel*: S-phase cells are depleted of ORC1b-GFP. Roots were labeled for 15 min with EdU (red), ORC1b-GFP (green) was detected by immunofluorescence and all nuclei were stained with DAPI (blue). The arrow marks a cell entering S-phase (EdU + ) that contains a reduced amount of ORC1b-GFP. The asterisk indicates a cell in S-phase (EdU + ) fully depleted of ORC1b-GFP. *Lower panel*: EdU versus ORC1b-GFP signal intensities in nuclei located in the proliferation zone (*n* = 137; a.u.: arbitrary units; Supplementary Data 1). **f** Live imaging showing the degradation of ORC1b-GFP upon S-phase initiation. The images illustrate cells showing the rapid decrease in ORC1b-GFP content (left panel). The GFP intensity during ORC1b-GFP degradation is shown as representative examples (*n* = 6) at the indicated time points (right panel; a.u.: arbitrary units; Supplementary Data 1). **g** *Left panel*: Cells undergoing the endocycle S-phase are depleted of ORC1b. Roots of plants expressing ORC1b-GFP were labeled for 15 min with EdU (red). Nuclei were stained with DAPI (blue). Arrows mark EdU + (S-phase) a cell without GFP signal. In the upper panel the white box shows the location of the endocycling cells analyzed in the transition zone and enlarged in the lower panels. *Right panel*: EdU versus ORC1b-GFP signal intensities in endocycling nuclei located in the transition and elongation zones (*n* = 372; a.u.: arbitrary units; Supplementary Data 1). Scale bars = 10 µm.

The patchy pattern of ORC1b protein in proliferating cells suggests that expression of the protein is regulated during the cell cycle. To determine precisely its dynamics, we used live imaging using ORC1b-GFP expressing plants. First, we studied the kinetics of accumulation of ORC1b and observed that cells progressing into mitosis were all expressing ORC1b from prophase to late telophase (Fig. 2a and Supplementary Movie 1). Nonetheless, based on the time covered by this experiment (a few hours) it is conceivable that ORC1b starts to be synthesized in G2 cells and accumulates before entering mitosis, as recently shown for human Orc1^23^. We confirmed this by a double-labeling strategy first with BrdU (15 min), and after a 2 h chase, a second labeling with EdU (15 min), which allows the identification of G2 nuclei as those labeled with BrdU but not with EdU (Fig. 2b). To define when ORC1b is synthesized in G2 we analyzed ORC1b-mRFP localization in plants expressing the G2/prophase/metaphase marker CYCB1;1-GFP^24,25^. We found that cells expressing high levels of CYCB1;1-GFP contained low amounts of ORC1b-mRFP, confirming that ORC1b started to accumulate in mid/late G2 (Fig. 2c). Live imaging revealed that full loading of ORC1b into chromatin occurs during the next G1, reaching maximum level 115–130 min after telophase (Fig. 2d and Supplementary Movie 2, arrowheads). The level of ORC1b in cells that just finished telophase was ~25% the maximum level detected in homogenously labeled nuclei.

### ORC1b is rapidly degraded shortly after the G1/S transition

To determine the ORC1b status during S-phase we analyzed the presence of ORC1b-GFP in cells labeled with a 15 min EdU pulse. We found that the vast majority of RAM cells (>88%) that are in S-phase (EdU positive) did not contain any detectable ORC1b-GFP signal (Fig. 2e and Supplementary Data 1). This indicates that ORC1b is not present during S-phase and suggests that it is degraded shortly after the G1/S transition. Live imaging experiments confirmed this since all the ORC1b-GFP signal of nuclei with maximum fluorescence fully disappeared in ~27–30 min (Fig. 2f and Supplementary Data 1 and Supplementary Movie 2, arrows). Since ORC1b is also expressed in the endoreplication domain of the root we investigated its dynamics in relation to S-phase during the endocycle. We found that after a 15 min pulse with EdU, ORC1b was excluded from most EdU positive (>82%), endoreplicating cells (Fig. 2g and Supplementary Data 1). This showed that ORC1b protein appears to be degraded also during S-phase of endoreplicating cells.

**Fig. 3:**
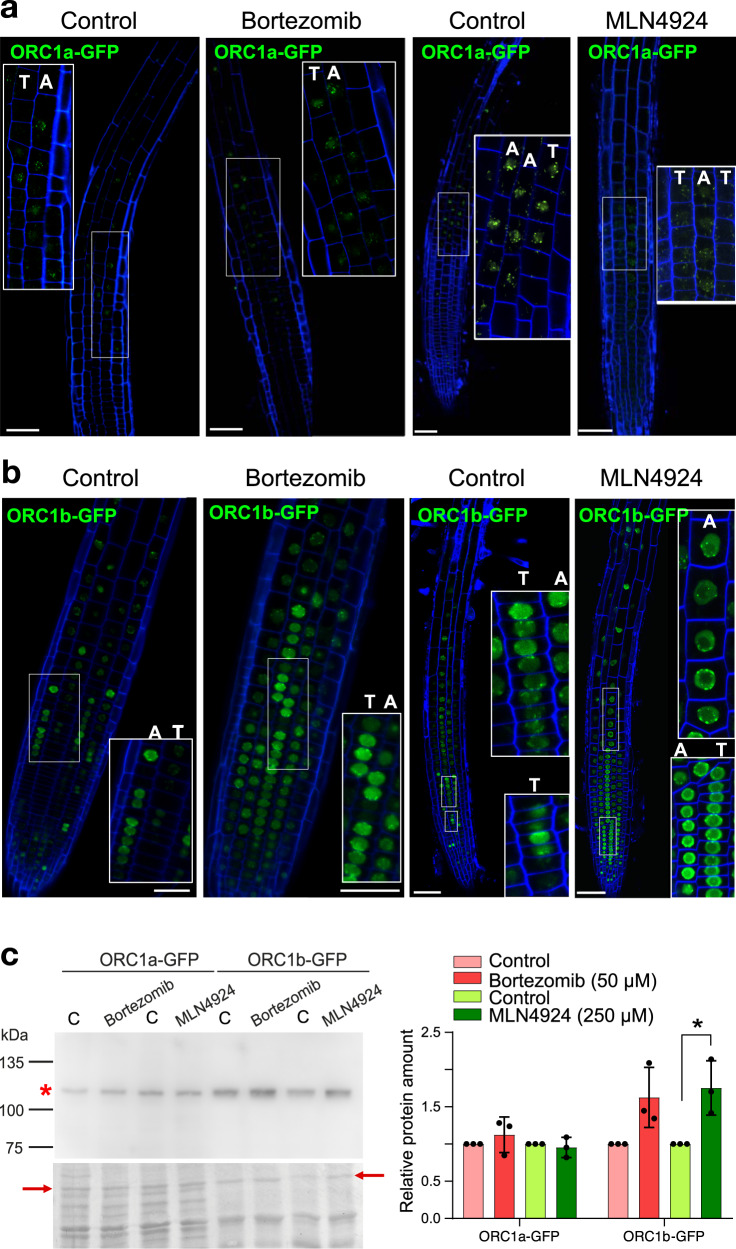
ORC1b is targeted for ubiquitin-mediated proteasome degradation. The expression of ORC1a-GFP (**a**) and ORC1b-GFP (**b**) was assessed by confocal microscopy after treatment with proteasome inhibitors, as indicated. Seven day-old roots were treated with bortezomib (50 µM, 4 h) or with MLN4924 (250 µM, 6 h). Details of ORC1a-GFP and ORC1b-GFP expression appear in the insets. Plasma membrane was stained with FM4-64 (blue). In all cases, confocal images were taken from the epidermal layer that contain trichoblasts (T) and atrichoblasts (A), as indicated, an only from the central layers in the image to avoid problems associated with the positions of cells outside the central plane. In the case of ORC1a (panel **a**) the analysis was restricted to the transition zone, where ORC1a is preferentially expressed. The number of roots analyzed was: ORC1a-GFP control (*n* = 11), ORC1a-GFP + bortezomib (*n* = 19), ORC1a-GFP + MLN4924 (*n* = 6), ORC1b-GFP control (*n* = 7), ORC1b-GFP + bortezomib (*n* = 6), ORC1b-GFP + MLN4924 (*n* = 6). Scale bars = 50 µm. **c** Analysis of protein levels with (as indicated) and without (C, control) proteasome inhibitors by Western blot (left panel). Asterisk points to the ORC1-GFP protein band. Loading controls are shown at the bottom. Arrows point to the bands used for normalization. Note that the protein pattern is different because seedlings were used for ORC1b and roots for ORC1a, based on their expression pattern. Western blots (*n* = 3 replicates from 2 independent protein extractions; Supplementary Data 1) were quantified and analyzed according to a one-way ANOVA analysis with the Kruskal–Wallis test and Dunn’s multiple comparisons (*p* < 0.05). Asterisk indicates statistically significant differences (*p* = 0.017).

To evaluate whether ORC1 proteins are targeted for proteasome degradation we first treated the GFP-tagged expressing plants with the proteasome inhibitor bortezomib and found that the amount of ORC1a and its subcellular localization pattern did not change (Fig. 3a). To avoid any possible cell type-related differences, we focused in the epidermal layer in the root zone where ORC1a is expressed. On the contrary, ORC1b accumulated to high levels in RAM cells (Fig. 3b). Likewise, MLN4924, a drug that selectively inhibits neddylation of all CULLIN1 (CUL1) RING ligases (CRL), also efficiently stabilized ORC1b protein (Fig. 3b), without having any significant effect on ORC1a (Fig. 3a). Evaluating the effect of these treatments with proteasome and CRL inhibitors by Western blotting statistically confirmed the results obtained by the treatment with MLN4924 in the confocal microscopy experiments (Fig. 3c and Supplementary Data 1). These results, together with live-imaging and EdU-labeling results, suggest that ORC1b, but not ORC1a, is an unstable protein being targeted by a CRL-type ubiquitin E3 ligase for proteasome degradation soon after the G1/S transition.

### Role of ORC1b in genome stability and of ORC1a in heterochromatin maintenance

The differences in expression domains of ORC1a and ORC1b, in the dynamics of their subnuclear distribution during the cell cycle and their stability strongly suggested that they play distinct roles. To investigate their functions, we identified T-DNA insertion mutant alleles, *orc1a-2* and *orc1b-2*, both of which do not produce full-length mRNAs (Supplementary Fig. 1). First, we analyzed the effects of these mutations on the RAM organization. We found that the size of root meristem cortical cells and the number of cortical cells were undistinguishable in wild type, *orc1a-2* and *orc1b-2* roots (Supplementary Fig. 2). These results suggest that loss or reduction of either ORC1a or ORC1b does not lead to obvious phenotypic changes of root development under standard growth conditions.

**Fig. 4:**
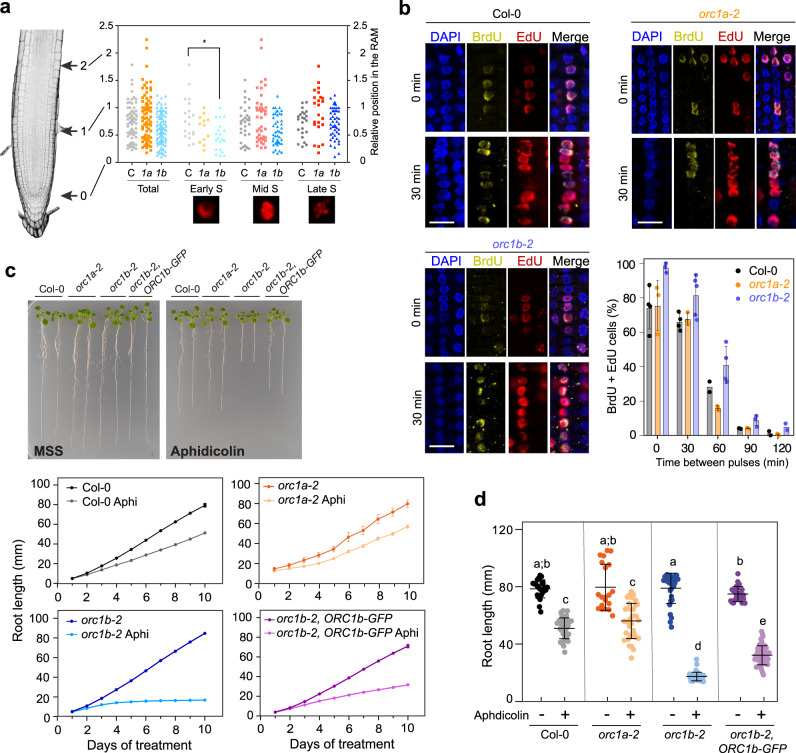
ORC1b is required for S-phase progression under normal and DNA replication stress conditions. **a** Position of S-phase cells along the root apex in wild type (C) and *orc1a-2* (*1a*) and *orc1b-2* (*1b*) mutants. S-phase nuclei were labeled with a 15 min pulse of EdU to allow the identification of early, mid and late DNA replication patterns. Position 0 corresponds to the QC and position 1 to the root apical meristem boundary. Total number of nuclei scored was 75 (*n* = 2 roots), 78 (*n* = 4 roots) and 116 (*n* = 4 roots) for Col-0, *orc1a-2* and *orc1b-2*, respectively (Supplementary Data 1). The asterisk indicates statistically significant differences between wild type and *orc1b-2* mutant (*p* value = 0.0054) using a one-way ANOVA test (*p* < 0.05). **b** S-phase progression was followed using a double-labeling strategy (a first 15 min BrdU pulse, a thymidine pulse of increasing time and a second 15 min EdU pulse). Examples of wild type Col-0, *orc1a-2* and *orc1b-2* nuclei are shown. S-phase progression, measured as the decrease in colabeling with the two pulses with increasing thymidine chase times, is also shown. For each chase time point the percentage of colabeled nuclei in each genotype was quantified. At least, 100 nuclei were scored for each time point from 2–4 root in each case. Bars in the plot are the mean values±standard deviation (Supplementary Data 1). Scale bars = 10 µm. **c** Effect of DNA replication stress produced by treatments with aphidicolin (Aphi) on root growth of wild type (Col-0), *orc1* mutants and *orc1b-2,ORC1b-GFP* expressing plants. Seedlings (3 day-old) were transferred to plates containing either normal medium or medium supplemented with aphidicolin (12 µg/ml), as indicated. A representative example of root growth on normal medium (MSS) or medium supplemented with aphidicolin at day 10 is presented (**c**, upper panel). Root length (mean ± s.e.m.) was determined every 24 h for a total of 10 days (**c**, lower panel) (*n* ≥ 20 roots/genotype; the precise value of *n* for each genotype and condition is provided in Supplementary Data 1). **d** Graph summarizing root length (mean ± s.d.) at the end of the experimental design (day 10) scored for all genotypes and conditions tested (the number of roots for each genotype and condition was as in panel **c**, and is detailed in Supplementary Data 1). Different letters indicate statistically significant differences applying a two-way ANOVA test (*p* < 0.05). Individual *p*-values are detailed in Supplementary Data 1.

Based on the canonical role of ORC1 in the activation of DNA replication origins, we sought to investigate possible effects of its depletion on S-phase progression. EdU labeling allows the identification of early, mid and late S-phase nuclei based on their staining patterns^26,27^, as slightly or punctate labeled, homogeneously and heavily labeled, and with apparent chromocenters, respectively (Fig. 4a). We found that in the *orc1b-2* mutant nuclei the early S-phase pattern was reduced significantly, suggesting problems of these cells to progress at the initial stages of S-phase (Fig. 4a and Supplementary Data 1). To evaluate S-phase progression we used a double-labeling strategy by labeling first with a 15 min pulse of BrdU, then chasing the cells for different times with thymidine and finally labeling with a second 15 min pulse of EdU (Supplementary Fig. 3). When the two labeling pulses are consecutive, most cells would appear colabeled. The colabeling percentage will progressively decrease by increasing chasing times between the BrdU and EdU pulses, depending on the number of cells finishing the S-phase before the second pulse. Thus, measuring the fraction of nuclei with the two labels after different chasing times between the two pulses provides a useful indication of S-phase progression. The chase time between pulses when the percentage of colabeling reaches 0 corresponds to the S-phase duration. When the two labeling pulses are consecutive (0 min between pulses), nuclei with the two labels averaged 74.2 ± 12.2% and 75.5 ± 14.6% in wild type and *orc1a-2* plants, respectively, indicating that the remaining cells had progressed out of S-phase when the second labeling pulse was initiated. However, in the case of the *orc1b-2* mutant, a larger fraction of nuclei (97.6 ± 2.8%) showed colocalization of the two labels, suggesting a delayed S-phase initiation or progression (Fig. 4b and Supplementary Data 1). Increasing the chasing time between the BrdU and EdU pulses allowed to conclude that S-phase progression was delayed in the *orc1b-2* mutant compared to the wild type and *orc1a-2* mutant plants (Fig. 4b and Supplementary Data 1).

Then we investigated whether the impairment of *orc1b-2* in S-phase initiation and progression is enhanced under DNA replication stress. Treatment of 3-day old seedlings with hydroxyurea (HU) or aphidicolin (Aphi), which slows down or arrest DNA replication forks, respectively, had very different consequences on root growth. While the sensitivity of *orc1a-2* to these treatments was similar to that of the wild type, aphidicolin (Fig. 4c and Supplementary Data 1), but not HU (Supplementary Fig. 4), produced a severe growth arrest of the *orc1b-2* mutant root. Comparison of the root length after 10 days of treatment of the various genotypes confirmed that differences with the Col-0 root growth were statistically significant for the *orc1b-2* mutant (Fig. 4d and Supplementary Data 1). It is worth noting that the presence of a detectable phenotype in the *orc1b-2* mutant indicates that the mutation is not complemented by the endogenous ORC1a protein present in the *orc1b-2* mutant. Expression of ORC1b-GFP in the *orc1b-2* mutant background partially restored root growth (Fig. 4d and Supplementary Data 1). This result was not due to defects in triggering the G2 DNA damage checkpoint as revealed by the upregulation of *RAD51* and *BRCA1* that was comparable in wild type, *orc1a-2*, *orc1b-2* and *orc1b-2,ORC1b-GFP* genotypes (Supplementary Fig. 5). It is conceivable that the defect in S-phase progression of the *orc1b-2* mutant is enhanced when additional pre-RCs need to be activated as a consequence of DNA replication fork arrest by aphidicolin.

**Fig. 5:**
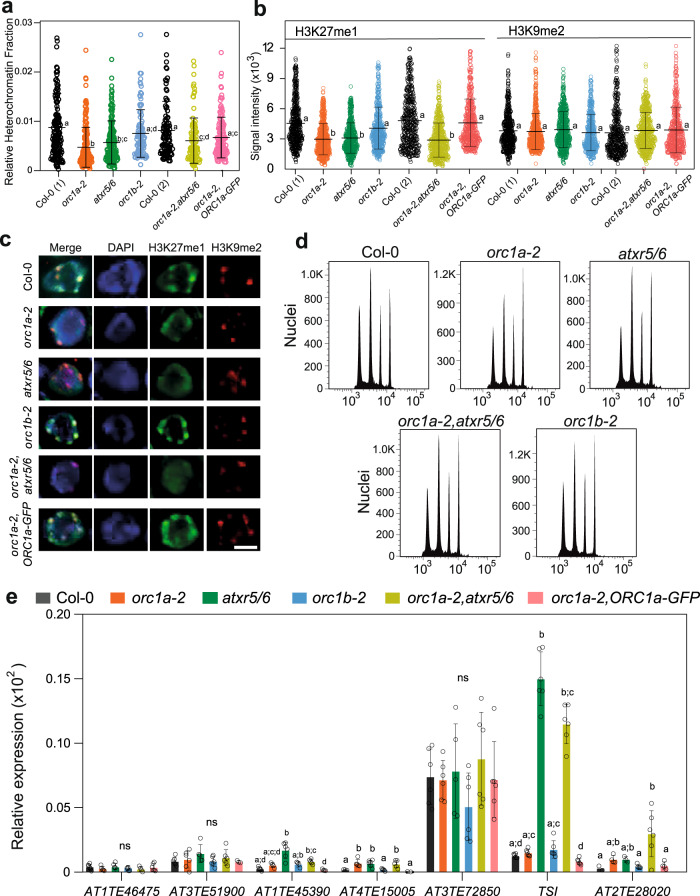
Role of ORC1a in the maintenance of H3K27me1 mark in heterochromatin. **a** Determination of the relative heterochromatin fraction (RHF) in nuclei of the indicated genotypes. Measurements were carried out in DAPI-stained nuclei (*n* ≥ 100 nuclei of *n* ≥ 6 roots per genotype; the precise value of n for each genotype is provided in Supplementary Data 1). Data reported are the mean ± s.d. Different letters near the average of each distribution indicate statistically significant differences according to a one-way ANOVA analysis with the Tukey’s multiple comparisons (*p* < 0.05). Exact *p*-values are detailed in Supplementary Data 1. **b** Distribution of H3K27me1 and H3K9me2 intensity signals detected in individual chromocenters of wild type (Col-0; two independent measurements), *orc1a-2*, *atxr5/6*, *orc1b-2*, *orc1a-2,atxr5/6* and *orc1a-2,ORC1a-GFP* nuclei. H3K27me1 and H3K9me2 were detected by immunofluorescence in endoreplicating root cells of 7 day-old seedlings of the indicated genotypes. Nuclei of epidermal cells were scored in all cases (Supplementary Data 1). Data reported are the mean ± s.d. Different letters near the average of each distribution indicate statistically significant differences between genotypes according to a one-way ANOVA analysis with the Tukey’s multiple comparisons. (*p* < 0.001). Exact p-values are detailed in Supplementary Data 1. **c** Examples of the identification of H3K27me1 and H3K9me2 by immunostaining (*n* = 3 immunodetection experiments, *n* ≥ 5 roots/experiments/genotype) in DAPI-stained root nuclei of the indicated genotypes. Scale bars = 5 µm. **d** Flow cytometry profiles of nuclei isolated from roots of the indicated genotypes. Note that all of them look very similar to that of Col-0, including the *atxr5/6* double mutant. **e** Quantification of TE reactivation by RT-qPCR in roots of Col-0 and the indicated genotypes (*n* = 2 biological independent samples with 3 technical replicates; Supplementary Data 1). Data reported are the mean ± s.d. Different letters indicate statistically significant differences according to a one-way ANOVA analysis with the Kruskal–Wallis test and Dunn’s multiple comparisons (*p* < 0.05). Exact *p*-values are detailed in Supplementary Data 1, ns not significant.

Given the preferential localization of ORC1a to the chromocenters we reasoned that it could play a role in heterochromatin organization. We calculated the relative heterochromatin fraction (RHF) by measuring the fraction of the nucleus covered by chromocenters in DAPI-stained nuclei, as defined earlier^28,29^. We found that *orc1a-2*, *atxr5/6* and *orc1a-2,atxr5/6* showed a reduction in their RHF, whereas *orc1b-2* and *orc1a-2,ORC1a-GFP* plants showed no significant differences to the wild type (Fig. 5a and Supplementary Data 1).

To gain a detailed insight into the possible defect of *orc1a-2* nuclei, we determined by immunofluorescence the level of H3K27me1 and H3K9me2, two characteristic plant heterochromatic histone marks^30^. We restricted our analysis to the nuclei of epidermal cells to avoid possible differences related to cell type or the different depth of the cell layers in the root. Also, we scored only nuclei located in the root region between 0.7 and 2.0 RAM units, where ORC1a is largely expressed. We used as control the *atxr5/6* double mutant, defective in the methyltransferases responsible for H3K27me1 deposition^31^. Quantification of both signals in immunofluorescence preparations revealed that the H3K27me1 signal was reduced in the *orc1a-2* mutant, a pattern that was comparable to that of the *atxr5/6* mutant (Fig. 5b, left group as indicated, and Supplementary Data 1). In contrast, the H3K27me1 distribution of the *orc1b-2* mutant was undistinguishable from the wild type Col-0 (Fig. 5b, left group as indicated, and Supplementary Data 1). These results indicate that ORC1a, but not ORC1b, is required for proper deposition and/or maintenance of H3K27me1. We also analyzed the triple *orc1a-2, atxr5/6* mutant, which showed the same H3K27me1 reduction than either the *orc1a-2* or the *atxr5/6* mutants, separately (Fig. 5b, left group as indicated, and Supplementary Data 1), indicating that ORC1a and ATXR5/6 are necessary for the dynamics of the heterochromatic H3K27me1 mark. Furthermore, the reduction of H3K27me1 signal was complemented by expressing ORC1a-GFP under its endogenous promoter (Fig. 5b, left group as indicated, Supplementary Data 1). In all cases the H3K9me2 signal, which did not change significantly in the genotypes studied, served as internal control (Fig. 5b, right group as indicated, and Supplementary Data 1; Supplementary Fig. 6). Examples of H3K27me1 and H3K9me2 immunodetection of DAPI-stained nuclei of the different genotypes studied are shown in Fig. 5c.

Mutations in the *ATXR5/6* genes are associated with re-replication defects^32^. Although we confirmed this phenotype in leaves #3/4 of 4-week-old seedlings of the *atxr5/6* double mutant, neither *orc1a-2* nor *orc1b-2* leaves showed this defect (Supplementary Fig. 7). Analysis of ploidy profiles in roots revealed that neither *orc1a-2* and *orc1b-2* nor *atxr5/6* mutations led to a re-replication phenotype (Fig. 5d), consistent with recent reports of a lack of this phenotype in *atxr5/6* root nuclei^33^. Transposon element (TE) reactivation is also a typical feature of the *atxr5/6* mutant^32^. We selected a subset of TEs based on their reduced H3K27me1 level in the atxr5/6 mutant to evaluate their expression in roots of the *orc1a-2* mutant^34^. Relative expression was not different for most TEs, except for Tsi, in roots of the *atxr5/6* mutant whereas in roots of the *orc1a-2* mutant only AT4TE15005 and AT2TE28020 showed a significant, but very small, reactivation (Fig. 5e and Supplementary Data 1). This reveals that reduction of H3K27me1 level in roots is not associated with significant TE reactivation in *orc1a-2* or *atxr5/6* roots, consistent with other reports^33^.

## Discussion

Several members of the pre-RCs are encoded by two genes in Arabidopsis, e.g. CDC6^12^, CDT1^13^, and ORC1^14^. Here we sought to investigate whether the two ORC1 proteins have distinct functional relevance in spite of their ~87% amino acid identity. The partially different expression domains of the two proteins strongly suggested that they could play different roles in developing organs. ORC1b is present in both proliferating and endoreplicating cells whereas ORC1a is restricted to endoreplicating cells. The nuclear pattern of ORC1b expression in proliferating cells was indicative of a strict cell cycle regulation. Consistent with this notion, *ORC1b* is a known E2F target^14^, as it is also the case for other key components of the pre-RCs including CDC6a, CDC6b, CDT1a, CDT1b, ORC1,2,3,4,6 and MCM2-7^14,35–39^. The subcellular localization of ORC1b showed that it is present in both eu- and heterochromatin whereas ORC1a is largely restricted to heterochromatin, clearly visualized in chromocenters.

The expression pattern of ORC1b in proliferating cells and its dynamics clearly revealed that it shares many features in common with the so-called “Orc cycle”, as already defined for mammalian cells^18^. In particular, ORC1b synthesis and degradation are similar to that described for human Orc1^17,23,40,41^ but not to that of other mammals where it shows rather constant levels during the cell cycle^42–44^. Thus, we can define the following Arabidopsis “ORC1b-cycle” as follows (Fig. 2): it starts to accumulate in mid G2 when CYCB1;1 levels are high, as reported recently for human Orc1^23^, reaching maximum ORC1b loading ~115–130 min after initiation of G1. Based on EdU labeling and live imaging we conclude that ORC1b is almost completely excluded from S-phase nuclei, because it is rapidly targeted for proteasome-mediated degradation upon S-phase entry.

The overall growth phenotype of *orc1b-2* mutant seedlings under normal conditions is similar to the wild type whereas it is hypersensitive to aphidicolin, but not to HU, a condition that is not very frequent in plants^45^. Mutations in human Orc1, as well as in other members of the pre-RCs (Orc4, Orc6, Cdc6, Cdt1), are at the molecular basis of the Meier-Gorlin syndrome^46–48^. In fact, patients carrying mutations in Orc1 exhibit the most drastic symptoms^49,50^ as a result of licensing defects of DNA replication origins. Moreover, in the absence of Orc1 cells could grow normally but then Cdc6 becomes essential for DNA replication and survival^51^. Thus, one possibility is that the lack of ORC1b in Arabidopsis increases S-phase duration because the amount of licensed pre-RC is reduced as a consequence of ORC1b defect, but it still seems sufficient to replicate the genome. Thus, after DNA replication stress produced by aphidicolin, DNA replication forks arrest and new DNA replication origins need to be activated to complete genome replication. Given the common evolutionary origin of ORC1b and CDC6^11^, CDC6 could partially compensate for the absence of ORC1b in the absence of DNA replication stress, as it occurs in human cells^51^. However, this may not be sufficient in the *orc1b-2* mutant after a treatment with aphidicolin, thus becoming hypersensitive compared to controls and leading to impaired root growth.

The function of the highly similar ORC1a protein is completely different to that of ORC1b protein since it does not seem to play a major role in DNA replication. Instead, cells lacking ORC1a possess defects in H3K27me1 deposition at the chromocenters, in particular in endoreplicating cells. The H3K27me1 plant-specific heterochromatic mark is deposited by the ATXR5 and ATXR6 methyltransferases in newly-replicated DNA through their PCNA-mediated association with DNA replication forks^31,52^. Mutations in the *ATXR5* and *ATXR6* genes lead to a re-replication phenotype particularly detected in endoreplicated nuclei of mature leaves by an abnormal accumulation of heterochromatic DNA and transposon reactivation^32^. Our results that the re-replication phenotype is not detected in root cells of the *atxr5/6* double mutant are consistent with recent findings of a high residual level of *ATXR6* expression in this mutant plant^33^. This may explain the lack of a re-replication phenotype of *orc1a-2* mutants, since ATXR5/6 and ORC1a are likely acting through the same pathway. Likewise, the lack of ORC1a in roots, where it is expressed, does not lead to a significant TE reactivation in the subset analyzed, although these TEs showed a reduced H3K27me1 level in the *atxr5/6* mutant^34^, consistent with recent reports^33^. An additional support comes from a recent report that the Orc1 protein of the yeast *T. delbrueckii* is required for heterochromatin formation but not for silencing^53^.

An attractive possibility is that ORC1a facilitates the access of ATXR5/6 to replicating heterochromatin during the endocycle. Additional support to a coupling between ORC1 and H3K27me1 maintenance comes from the known interaction of ORC1 with components of the TREX-2 complex^54^ that bind RNA polymerase II and is necessary to resolve R-loops formed by displacement of the non-transcribed DNA strand^55^. In fact, mutations in genes encoding members of the TREX-2 complex are suppressors of the re-replication and transposon reactivation phenotypes of *atxr5/6* mutants^56^. Therefore, it is conceivable that ORC1a could interact with the TREX-2 complex to facilitate recruitment of ATXR5/6 to the pre-RCs, which after initiation of DNA replication can be transferred to the replisome in a PCNA-dependent manner.

Our results are consistent with the view that plant ORC1a and ORC1b have acquired different functions in heterochromatin maintenance and DNA replication, respectively, whereas in animals a single protein plays a dual role. It seems that after partial genome duplication^57^, the parental *ORC1b* gene has retained the canonical function in DNA replication and the new *ORC1a* gene became specialized in heterochromatin maintenance in endoreplicating cells. Interestingly, the Orc1 gene has also duplicated during yeast evolution and one copy gave rise to the Sir3 gene of *S. cerevisiae*^58^. It is known that the yeast *Kluyveromyces lactis* diverged from *S. cerevisiae* prior to the *ORC1* gene duplication and that ORC1 plays a role with Sir2 and Sir4 proteins in heterochromatin maintenance.

There are also precedents in other eukaryotic systems that ORC1 binds heterochromatin-associated proteins such as Sir1 in *Saccharomyces cerevisiae*^59^ or HP1 in Drosophila, Xenopus and human cells^60–62^. A plausible hypothesis is that the BAH domain of Arabidopsis ORC1a participates in the spreading of the H3K27me1 heterochromatic mark. This is consistent with a similar role of *S. cerevisiae* Sir3 protein^63^. Therefore, the evolutionary history that occurred in the yeast lineage might have also taken place independently in Arabidopsis, where ORC1a and ORC1b proteins acquired a division of labor that did not occur in animals. This evolutionary history highlights the essential connection between DNA replication and heterochromatin maintenance to preserve genome stability.

## Methods

### Plant materials and growth conditions

*Arabidopsis thaliana* seeds (Col-0 ecotype) were stratified for 48 h and grown in 0.5x MS medium (pH 5.7) supplemented with MES (Sigma), vitamins (Duchefa) 0.5 or 1% sucrose (Duchefa), and 0.8 or 1% agar (Duchefa) in an incubator at 21 °C with 60% moisture, under long day conditions (16 h light, 8 h dark, fluorescent tubes Philips MASTER TLD Super80, 36 W, 4000 K, 100 µmol/m^2^/s). The T-DNA mutants used in this work were *orc1a-2* (WiscDsLox287F12) and *orc1b-2* (SALK_042536C). For their characterization, total genomic DNA was analyzed by PCR using Taq DNA polymerase (Biotools) and primers listed in Supplementary Table 1. MS medium was supplemented with antibiotics for plant selection and specific drugs according to the treatment required.

### Cloning and generation of transgenic plants

Genomic fragments containing the promoter and the coding region of *ORC1a* (At4g14700) and *ORC1b* (At4g12620) without STOP codon, were PCR amplified from F4C24 BAC^64^ or genomic DNA respectively using AccuPrime or Pfx Taq polymerase (ThermoFisher Scientific) with primers listed in Supplementary Table 1. PCR fragments were cloned into pDONOR221 (Invitrogen), sequences checked (Macrogen) and transferred to Gateway destination vectors: pGWB433 and pGWB533 for *ORC1b*- and *ORC1a*-GUS C-terminal fusion constructs respectively, pGWB450 and pGWB550 for *ORC1b*- and *ORC1a*-G3GFP C-terminal fusion constructs, respectively, and pGWB453 for *ORC1b-mRFP* C-terminal fusion construct^65^. Transgenic plants were generated by the floral dip method^66^ using the *Agrobacterium tumefaciens* C58C1 strain. Transformant seeds were selected in 0.8% agar MS plates containing either 15 µg/ml hygromycin (*ORC1a* transgenics) or 50 µg/ml kanamycin (*ORC1b* transgenics). Independent lines harboring one T-DNA insert were selected and showed similar expression patterns of the protein of interest. ORC1b-mRFP homozygous lines were crossed with the pCYCB1;1-GFP transgenic line, and the resulting T2 seeds were selected under the confocal microscope.

### Drug treatments

To study the regulation of the ORC1 proteins, GFP-tagged seedlings were incubated in liquid MS media containing different inhibitors. Plants (4 days post sowing; dps) were acclimated in the liquid MS media during ~12 h to recover from the temporary hypoxia. To inhibit the proteasome pathway plants were incubated with 50 µM bortezomib (Selleckchem) for 4 h or with 250 µM MLN4924 (APExBIO) for 6 h. Control plants were incubated in MS containing DMSO used as the drugs solvent. Incubations were carried out in the plant culture chamber. To assess root growth under DNA replication stress conditions, 3 dps *orc1* mutants, *orc1b-2,ORC1b-GFP* and Col-0 seeds were grown in 1% agar MSS plates and transferred to 1% agar MS plates containing 0.5% sucrose supplemented with 1 mM hydroxyurea (Sigma), 12 µg/ml aphidicolin (Sigma) or no drug as controls. Seeds that did not germinate were removed from the analysis. Root growth was measured every 24 h for a total of 10 days.

### Isolation of nuclear proteins for Western blot analysis

To evaluate the effects of proteasome inhibitors on the regulation of ORC1 proteins, nuclei were extracted from 7 dps whole seedlings (*ORC1b-GFP*) or roots (*ORC1a-GFP*) treated as indicated, using Honda Buffer (0.44 M sucrose, 1.25% Ficoll, 2.5% Dextran T40, 20 mM Hepes HOK pH7.4, 10 mM MgCl_2_, 0.5% Triton X-100). 70 μg of nuclear proteins were loaded in a 6% Tris-glycine polyacrylamide gels to run SDS-PAGE and subsequent Western Blot. The proteins were transferred to a membrane, blocked 5% non-fat milk and then incubated with the primary antibody overnight at 4 °C (anti-GFP (Abcam ab5450) diluted 1:2000). After three washes the membrane was incubated with the secondary antibody for 1 h at room temperature (Anti-goat IgG -Peroxidase (Sigma A-5420) diluted 1:10000), washed again three times and proteins were detected using the kit Immobilon WB Chemiluminescent for HRP substrates (Millipore).

### RNA extraction and RT-qPCR

Seedlings were frozen in liquid nitrogen and ground with glass beads using a Silamat S5 device (Ivoclar Vivadent) for 10 s. RNA was extracted using TRIzol reagent and treated with DNAse I according to manufacturer instructions (ThermoFisher Scientific). One µg of total RNA was used as template for reverse transcription using iScript Reverse Transcription Supermix as recommended by the manufacturer (BioRad). qPCR was performed with iTaq Universal SYBR®Green Supermix (BioRad) and primers listed in Supplementary Table 1, in a CFX384 machine (BioRad). Gene expression levels were compared to the reference gene *GAPC-2* (*At1g13440)*, *ACT2* (At3g18780) or *IPP2* (At3g02780).

### Histochemical detection of GUS activity

Detection of GUS activity was performed using 5-bromo-4-chloro-3-indoyl-β-D-glucuronide (X-Gluc) as described in^67^, with modifications^68^. Briefly, 4, 7 or 12 dps seedlings were infiltrated into fresh GUS substrate for 5 min (30,000 Pa) and then incubated for 72 h at 37 °C. Seedlings were, washed twice with water and preserved in 1x PBS-50% glycerol at 4 °C until observation under an Axioskop2 plus microscope (Zeiss) or a MZ9.5 stereomicroscope (Leica).

### Confocal microscopy and live imaging

Roots were stained either with 50 µg/ml propidium iodide (Sigma; for cell walls) or 10 µM FM4-64 (Life technologies; for membranes) and directly examined using LSM510 or LSM710 confocal microscopes (Zeiss). Immunostained roots were observed either using Zeiss LSM710, Zeiss LSM800 or Nikon A1R + confocal microscope. For live imaging, seedlings were grown for 3 days, transferred to P35 glass bottom dishes (MatTek) and the roots covered with a piece of 1% agar MS solid media. The dishes were hung vertically in the plant culture chamber for 24 h. In some cases, prior to confocal observation, 10 µM FM4-64 were injected under the solid media to stain the membranes. In vivo images were acquired every 2 or 3 min to observe rapid processes or every 15 min to supervise G1 progression, either manually or using the time lapse module. Images and movies were edited using FIJI^69^.

### Immunostaining

For immunostaining assays, 7 dps seedlings were fixed in 4% paraformaldehyde in microtubules stabilizing buffer (MTSB; 50 mM PIPES, pH 6.9, 5 mM EGTA, 5 mM MgSO_4_) for 20 min with vacuum infiltration (30,000 Pa). After several washes with MTSB, PBS and water, seedlings were placed on Superfrost plus slides (ThermoFisher Scientific) and air-dried overnight. Plant cell walls were partially digested with 20 mg/ml driselase^70^ (Sigma) in MTSB for 45 min at 37 °C and the slides were washed with PBS. Membranes were permeabilized with 10% DMSO, 3% Igepal CA-630 in MTSB for 1 h. Non-specific sites were blocked in 3% BSA, 10% Horse Serum (HS) in PBS for 1 h at 37 °C. Samples were incubated with primary antibodies overnight at 4 °C and with the secondary antibodies for 1 h at room temperature. Primary antibodies used were: anti-H3K9me2 (Abcam ab1220, 1:1000), anti-H3K27me1 (Millipore 07-448, 1:1000) and anti-GFP (Abcam ab5450, 1:2000). They were detected with secondary antibodies from ThermoFisher: Donkey anti-Rabbit IgG-Alexa Fluor 488 (A-21206), Donkey anti-Goat IgG-Alexa Fluor 488 (A-11055) and Donkey anti-Mouse IgG-Alexa Fluor 555 (A-31570) diluted 1:500. Nuclei were counterstained with 10 μg/ml DAPI (Merck) and slides mounted in Mowiol 4-88 (Sigma). The combinations of primary and secondary antibodies were specific for each experiment.

### Assessing progression through S-phase

To track cell cycle progression either 7 dps Col-0 wild type, GFP-tagged line or *orc1* mutant plants were used. Nuclei undergoing S-phase were labeled with thymidine analogs either EdU (5-ethynyl-2-deoxyuridine; Life Technologies) or BrdU (5-bromo-2-deoxyuridine; Sigma). All the incubations were performed in liquid MS, at room temperature and protecting the samples from light to avoid the degradation of the analogs. Detection of S-phase nuclei was carried out with a single 15 min pulse of 200 μM EdU. For the analysis of S-phase progression a combination of two pulses was used. First, a 15 min pulse with 200 μM BrdU was done. Then, the analog was washed off and the seedlings were incubated with 200 μM thymidine (Sigma) during increasing chase times to allow cell cycle progression. After the chase time, a second pulse with 200 μM EdU was done. Roots were then processed as described for the immunostaining protocol. After immunodetection of the GFP in the tagged line (primary antibody: anti-GFP (ThermoFisher A6455; 1:2000) and secondary antibody: Goat anti-Rabbit – Alexa Fluor 488; (ThermoFisher A-11034; 1:500)), EdU was detected using Click-iT EdU Alexa Fluor 647 Imaging kit (ThermoFisher), following manufacturer´s instructions for 30 min at room temperature. Prior to immunodetection of BrdU, DNA was relaxed through a mild digestion by incubating with 0.005 U/μl DNase I RNase free (Roche) for 1 h 30 min at 37 °C. DNase I was inactivated with several washes of ice-cold 8 mM EDTA-PBS. BrdU was immunodetected using anti-BrdU (Becton Dickinson, 347580) diluted 1:200 and Donkey anti-Mouse – Alexa Fluor 555 (ThermoFisher A-31570) diluted 1:500.

### Positioning of cells along the root

Since the end of the meristem for each cell file is not the same, the meristem size was determined in every cell file of the epidermis. To do so, the distance from the QC to the first cell in focus in the epidermis was measured as well as the length of all other cells in the file. The end of the meristem was considered as the first elongated cell^71^. The relative position of each cell was calculated by normalization to the length of the RAM in each cell file. Thus, in this analysis, the cell marking the end of the meristem will be at position 1 and 0 corresponds to the QC. The relative position was calculated according to:

$$	{{{{{\rm{Relative}}}}}}\; {{{{{\rm{position}}}}}}\\ 	=\frac{\left({{{{{\rm{Cell}}}}}}\,{{{{{\rm{length}}}}}}+{{{{{\rm{Initial}}}}}}\; {{{{{\rm{distance}}}}}}\; {{{{{\rm{from}}}}}}\; {{{{{\rm{the}}}}}}\; {{{{{\rm{QC}}}}}}\right)}{\left({{{{{\rm{Length}}}}}}\; {{{{{\rm{of}}}}}}\; {{{{{\rm{cell}}}}}}\; {{{{{\rm{determining}}}}}}\; {{{{{\rm{the}}}}}}\; {{{{{\rm{end}}}}}}\; {{{{{\rm{of}}}}}}\; {{{{{\rm{the}}}}}}\; {{{{{\rm{meristem}}}}}}+{{{{{\rm{Initial}}}}}}\; {{{{{\rm{distance}}}}}}\; {{{{{\rm{from}}}}}}\; {{{{{\rm{the}}}}}}\; {{{{{\rm{QC}}}}}}\right)}$$

Epidermal T-clones and cells undergoing mitosis were removed from the analysis.

### Measurements of fluorescent intensity

Stack images of the roots (1 µm section) were acquired using a ×40 oil objective with a NIKON A1R + confocal microscope. Tile-scanning (4 × 1 tiles) was used to ensure the imaging of the whole meristem and the elongation zone of the roots. The Z Project tool (Projection type: Sum slices) was used to sum the fluorescence intensity of the pixels corresponding to each nucleus present in the epidermal layer. The background fluorescence was subtracted for each color channel and the fluorescent intensity of chromocenters and nuclei was measured as the integrity density of a determined ROI. In all cases, independent measurements were taken for each color channel. Data acquisition and processing were done using FIJI. The Relative Heterochromatin Fraction (RHF) was calculated using the formula RHF = [Ac × (Ʃ Ic − Ib)] / [An × (In − Ib)]. Ac: the total chromocenters area; Ic: fluorescence intensity of chromocenter; Ib: fluorescence intensity of the background; An: area of the nucleus; In: fluorescence intensity of the nucleus.

### Flow cytometry

Seeds from *orc1* mutants, *atxr5/6*, *orc1a-2,atxr5/6* and Col-0 plants were grown for 7 days in MS-agar plates or for 24 days in soil. Roots of 7 days old seedlings or leaves #3/4 of 24 days old plants of the indicated genotypes were chopped in 500 µL of cold Galbraith solution (20 mM MOPS, pH 7.0, 45 mM MgCl2, 30 mM sodium citrate, 0.1% Triton X-100, pH 7.0^72^) using a single edge razor blade in Petri dishes on ice. The released nuclei were filtered through a 30 μm nylon net filter and RNAse at 100 µg/ml and propidium iodide at 50 µg/ml were added. Nuclei were analyzed using a FACSCanto A flow cytometer (Becton Dickinson) and 10,000 events were measured. The experiments were repeated in triplicate and data were analyzed with FloJo software.

### Reporting summary

Further information on research design is available in the Nature Portfolio Reporting Summary linked to this article.

## Supplementary information

Supplementary Information

Peer Review File

Description of Additional Supplementary Files

Supplementary Data 1

Supplementary Movie 1

Supplementary Movie 2

Reporting Summary

## Data Availability

Authors declare that all data in this work are available within the article, its supplementary information or from the corresponding author. Source data are provided in Supplementary Data 1.
