# Supplementary material for: Distinct roles of Arabidopsis ORC1 proteins in DNA replication and heterochromatic H3K27me1 deposition
Source: Nat Commun. 2023 Mar 7;14:1270. doi: 10.1038/s41467-023-37024-8 (PMC9992703; doi:10.1038/s41467-023-37024-8)
Supplement: Supplementary file 1 — Supplementary Information [file 41467_2023_37024_MOESM1_ESM.pdf]

Supplementary Information

Distinct roles of Arabidopsis ORC1 proteins in DNA replication and heterochromatic H3K27me1 deposition

Zaida Vergara[1,*], María S. Gomez[1,*], Bénédicte Desvoyes[1], Joana Sequeira-Mendes[1], Kinda Masoud[2], Celina Costas[1,+], Sandra Noir[2], Elena Caro[1,$], V. Mora-Gil[1], Pascal Genschik[2], Crisanto Gutierrez[1,+]

1 Centro de Biología Molecular Severo Ochoa, CSIC-UAM, Nicolás Cabrera 1, Cantoblanco, 28049 Madrid, Spain

2 Institut de Biologie Moléculaire des Plantes, CNRS, Université de Strasbourg, 12 rue Général Zimmer, 67084 Strasbourg cedex, France

* These authors contribute equally

Key words: Origin recognition complex (ORC), ORC1, DNA replication, cell cycle, endoreplication, heterochromatin, epigenetics, genome instability, Arabidopsis

Running title: ORC1, genome stability and heterochromatin

+ Correspondence could be addressed to cgutierrez@cbm.csic.es

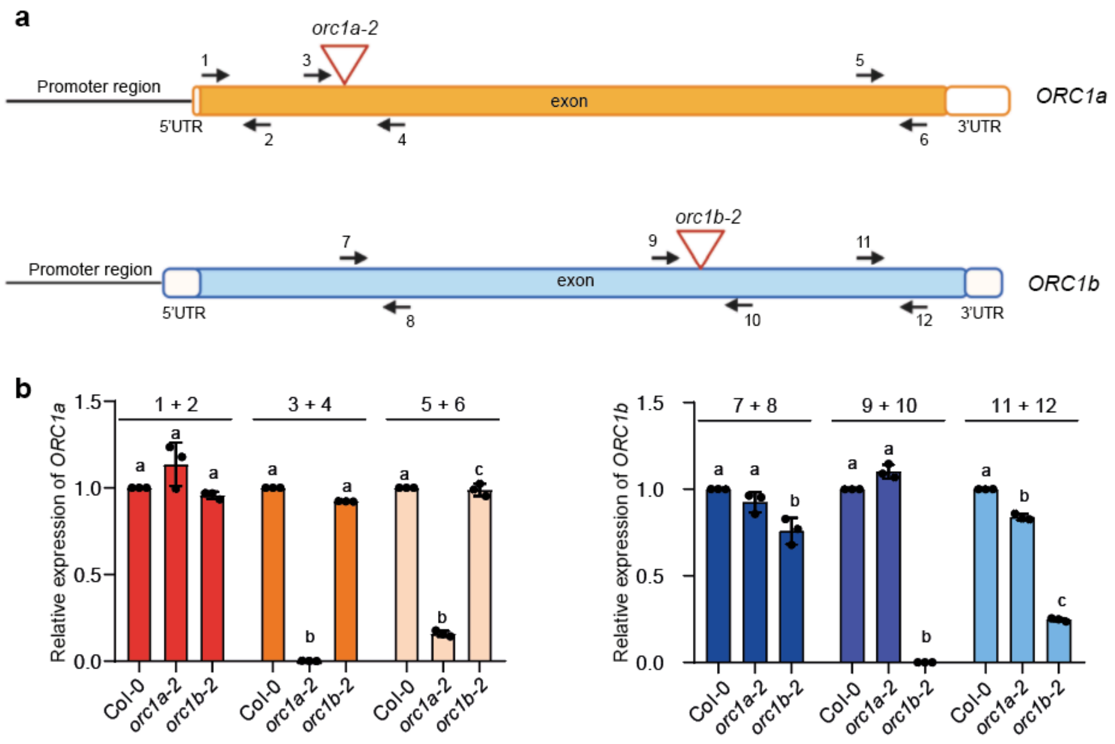

Supplementary Fig. 1. Description of *orc1* T-DNA insertion mutants used in this study. a, Schematic representation of *ORC1a* (orange) and *ORC1b* (blue) genes, the T-DNA insertions (red triangles) and the primers (arrows) used to evaluate the mRNA levels. **b,** Detection of full-length mRNA levels in the wild type (Col-0) and *orc1* mutants with the indicated primer pairs (mean ± s.d.). The expression of *ACTIN2* (*ACT2*; AT3G18780) was used as reference gene, and primers used in each are indicated with numbers. Primer sequences are listed in Supplementary Table 1. One example, out of two independent experiments, is plotted (n = 3 technical replicates). Different letters indicate statistically significant differences between genotypes by applying a one-way ANOVA test with the Tukey's multiple comparisons ($p<0.05$).Exact p-values are detailed in Supplementary Data 1.

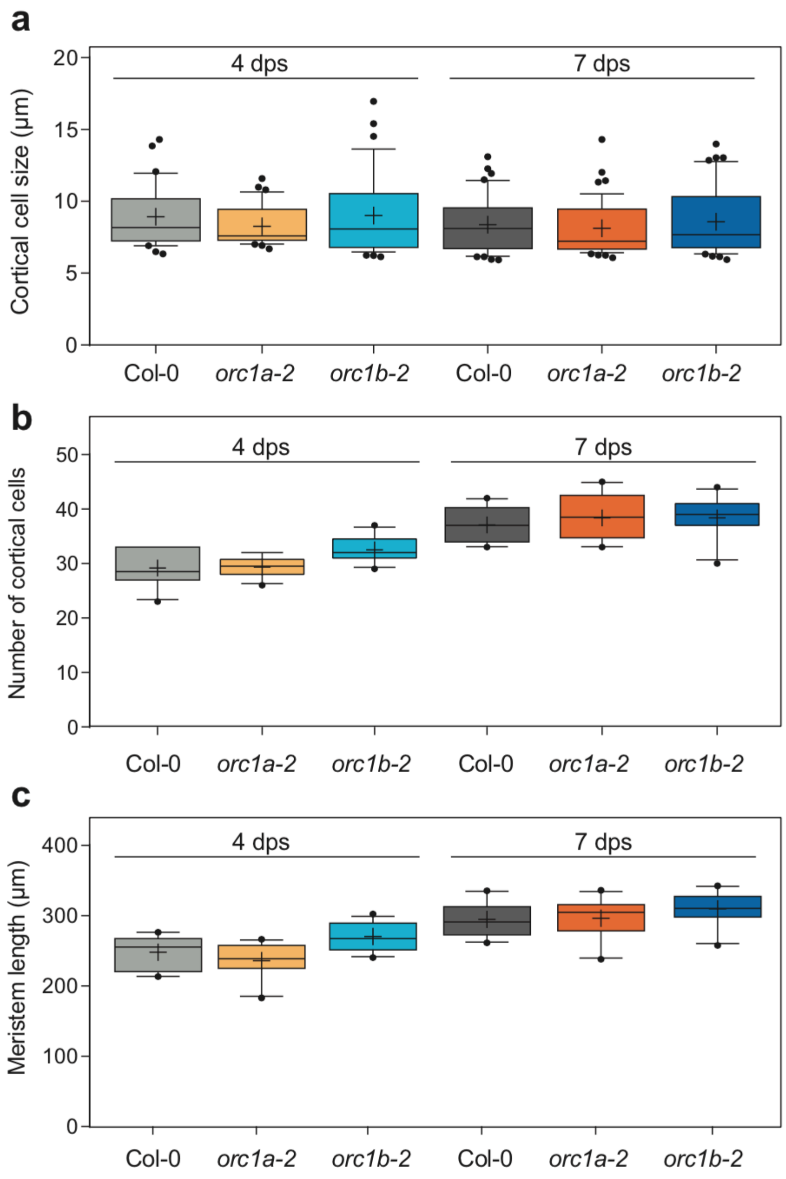

Supplementary Fig. 2. Root apical meristem parameters of wild type and *orc1* mutants. a, Cortical cell size. **b,** Cortical cell number. **c,** Meristem length. All measurements were carried in 4 day-old (n=12) and 7-day-old (n=10) root meristems, as indicated, in the three genotypes Col-0 (grey), *orc1a-2* (orange) and *orc1b-2* (blue). Although a trend can be observed towards a longer RAM with more cortical cells in the *orc1b-2* mutants, differences were not statistically different according to a one way ANOVA analysis with the Kruskal-Wallis test and Dunn's multiple comparisons. Line in the middle of the box is the median and the symbol + represents the mean. Whiskers are drawn to the 10th percentile up to the 90th percentile. Outliers are plotted (dots).

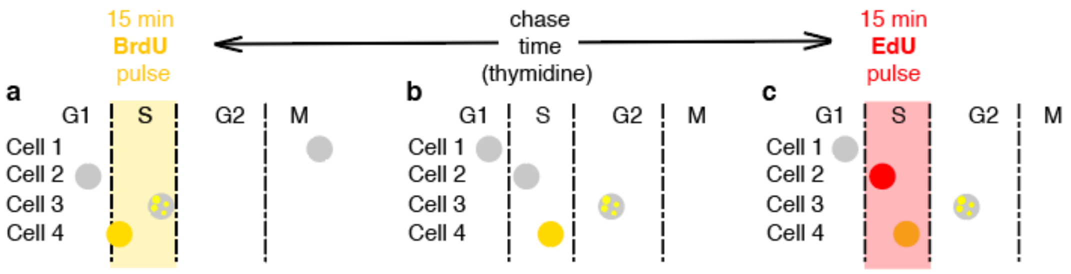

Supplementary Fig. 3. Double-labeling strategy to identify cells in G2 and S-phase progression. a, A population of root meristem cells is subjected to a 15 min pulse with BrdU (yellow). Here we provide an example of 4 cells of this population to illustrate the experiment. Cells 1 and 2 are outside S phase and therefore are not labeled with BrdU. Cells 3 and 4 are in different stages of S-phase and both appear labeled, homogenously (early/mid S) or with typical chromocenter labeling (late S). **b**, Then, a chase given in the presence of thymidine (to reduce BrdU incorporation), e.g. 2h in this example. During this time all cells progress through the cell cycle. **c**, A second 15 min pulse, now with EdU (red), is given to label cells in S-phase during the pulse. Cell 1, which was in mitosis, divided and is in G1. Consequently, it is not labeled either with the BrdU pulse or the EdU pulse. Cell 2, which was in G1 during the first pulse has now moved into S-phase and is labeled with EdU (detected as a red nucleus). Cell 3 which had a late S-phase BrdU labeling pattern has moved now to G2 and is not labeled with EdU. Finally, cell 4 which was in early S during the first BrdU pulse has progressed but still is in S-phase, thus becoming also labeled with the second EdU pulse (orange nucleus).
In summary, (i) cells exclusively labeled with the first pulse can be unequivocally identified as cells undergoing G2, (ii) those labeled exclusively with the second pulse were in G1 before the chase period and (iii) those with the two labels had been in S-phase between the first and second pulses.
This strategy can be used to measure progression and length of S-phase. Beginning with a consecutive labeling with the two analogs and increasing the chasing time between pulses, it is possible to score the percentage of cells colabeled at different times. Without any chasing time between the two pulses most cells are colabeled whereas this amount decreases with increasing chasing times between the BrdU and EdU pulses until it reaches 0. The chasing time when this occurs corresponds to the length of S-phase.

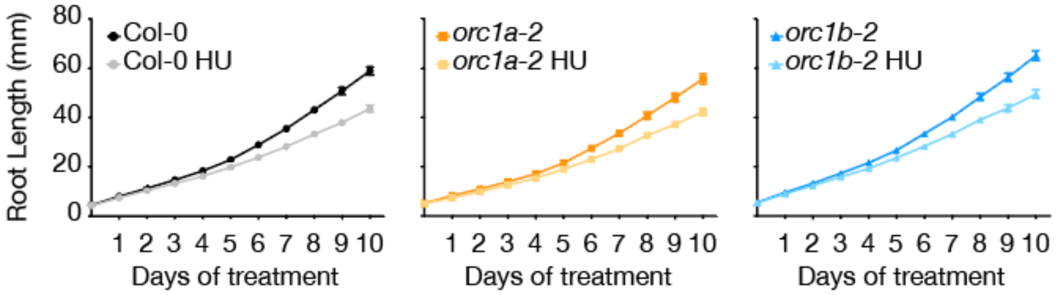

Supplementary Fig. 4. Response to hydroxyurea (HU) treatment of wild type, *orc1a-2* and *orc1b-2* mutants. Seedlings (3 day-old) were transferred to plates containing either normal medium or medium supplemented with hydroxyurea (HU; 1 mM). Root length (mean ± s.e.m.) was determined every 24h for a total of 10 days (n≥35 roots/genotype; the precise value of n for each genotype and condition is provided in Supplementary Data 1).

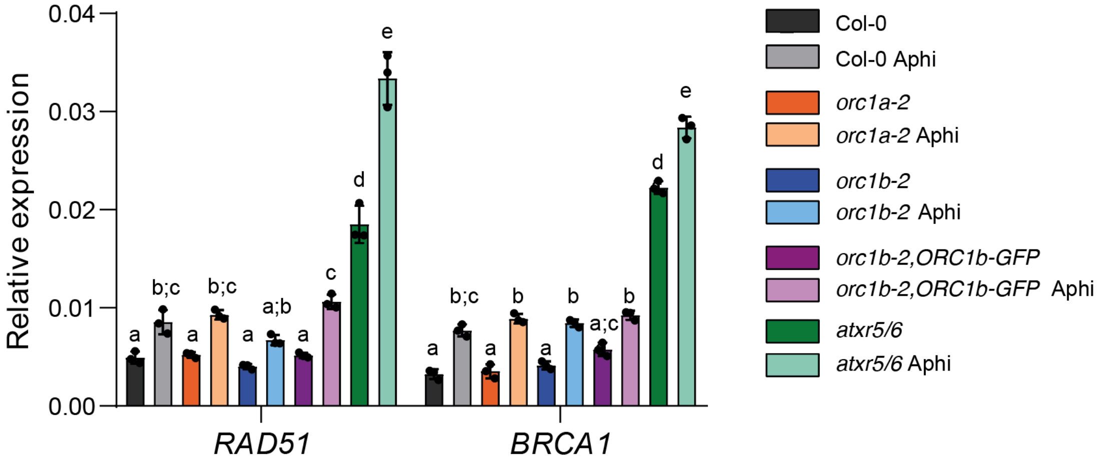

Supplementary Fig. 5. G2 checkpoint related genes in wild type (Col-0) and mutants, as indicated, with and without aphidicolin treatment. mRNA levels of *RAD51* and *BRCA1* genes in extracts of whole seedlings after 10 days of treatment with 12 µg/ml of aphidicolin. Expression levels (mean ± s.d.) were normalized against the reference gene GAPC2. One example, out of two independent experiments, is plotted (n = 3 technical replicates). Different letters near the average of each distribution indicate statistically significant differences between genotypes by applying a two-way ANOVA test ($p<0.05$). Exact p-values are detailed in Supplementary Data 1.

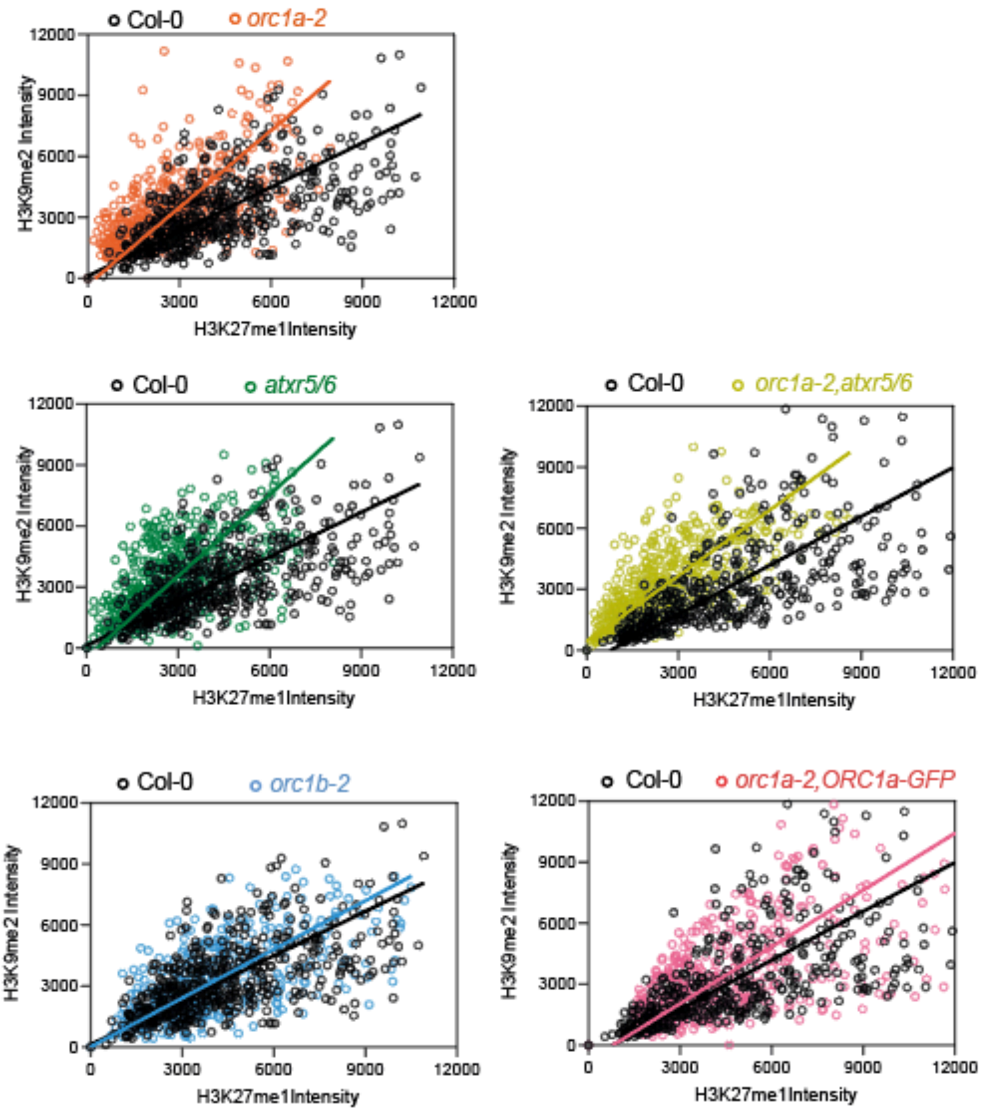

Supplementary Figure 6. Correlation of H3K27me1 and H3K9me2 signals in heterochromatin. Fluorescence intensity signals of both heterochromatin marks were plotted for individual chromocenters of wild type (Col-0, in black in all panels), *orc1a-2*, *atxr5/6*, *orc1b-2*, *orc1a-2,atxr5/6* and *orc1a-2,ORC1a-GFP* genotypes. Note that measurements for Col-0 come from two independent experiments, one for *orc1a-2*, *atxr5/6* and *orc1b-2*, and another for *orc1a-2,atxr5/6* and *orc1a-2,ORC1a-GFP*. The regression lines were generated using Deming (Model II) linear regression. Details on sample size and statistical analysis are summarized in Supplementary Table 1.

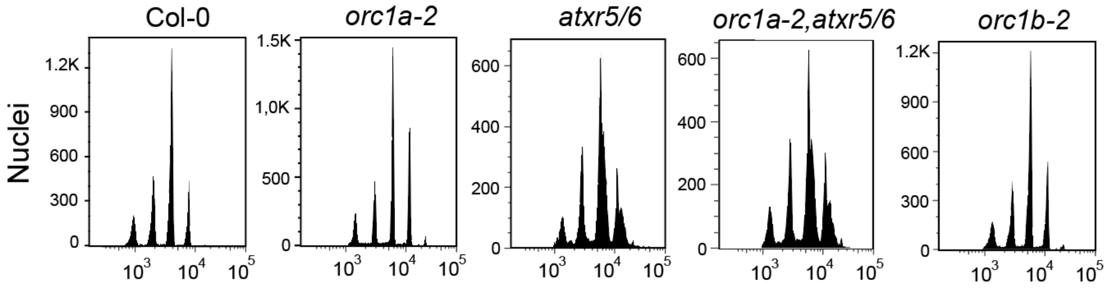

Supplementary Figure 7. Flow cytometry profiles of nuclei of the indicated genotypes isolated from leaves #3/4 of 4 week-old plants. Note the typical ploidy profile of the *atxr5/6* double mutant, also visible in the *orc1a-2,atxr5/6* triple mutant.

Supplementary Table 1. Primers used in this study.

Primers used for cloning.

Primer name	Sequence
**pORC1a(-1324)::O F**	GGGGACAAGTTTGTACAAAAAAGCAGGCTGGACCTCTAGTAGCATATGCC
**pORC1a(-87)::ORC**	GGGGACAAGTTTGTACAAAAAAGCAGGCTTCGTGCAAACATTTCCCGCC
**pORC1a::ORC1a F**	GGGGACCACTTTGTACAAGAAAGCTGGGTCCAAGTAATTGGCCAACCATG
**pORC1b(-793)::OF F**	GGGGACAAGTTTGTACAAAAAAGCAGGCTGTGGGTTTCCAGAGAGTGG
**pORC1b::ORC1b F**	GGGGACCACTTTGTACAAGAAAGCTGGGTCCAAGTAATTGGCCAGCCA

Primers used for the characterization of T-DNA insertion mutant lines.

Primer name	Sequence
**ATXR5 LP**	CCATTGGAACTTGGCTTTGTGTC
**ATXR5 RP**	AATAGGACCATCTGCTTCAACTGTG
**ATXR5 T-DNA**	TGGTTCACGTAGTGGGCCATCG
**ATXR6 LP**	AGCTTTGCTGGTTGTTTACCGGA
**ATXR6 RP**	CCATGTTGAGTAAATGTCGAAGAC
**ATXR6 T-DNA**	AACGTCCGCAATGTGTTATTAAGTTGTC

Primers used for qPCR analysis.

Primer name	Sequence
**GAPC2 F**	TCCAACGCTAGTTGCACCAC
**GAPC2 R**	TGGACAGTGGTCATGAGTCC
**BRCA1 F**	TGCATCCATTAAGTTGCCCTGTG
**BRCA1 R**	TAGGCTGAGAGTGCAGTGGTTC
**RAD51 F**	GCTAGTTCCTCTGGGGTTCA
**RAD51 R**	GGAACCAGTTTCAATACCTCCT
**IPP2 F**	TCCAGGACAATGCACTAGGTGTG
**IPP2 R**	AGGAGCCTTGTACAGCATACGTC
**AT1TE46475 F**	CAAATTTTGAAGGGACTCTCCAT
**AT1TE46475 R**	GCACATACTTGCAATGTCCACCA
**AT3TE51900 F**	TCATTGGGATGGAAACTTCTTC
**AT3TE51900 R**	AAAACGCTCTCCACTTTTCATC
**AT1TE45390 F**	CAAAGCTCAATCGAGTCGTAAA
**AT1TE45390 R**	TTGCTTGATGTTATGCTTTCAA
**AT4TE15005 F**	CTATACCCGAAGCCACAATCTC
**AT4TE15005 R**	GCAATCTCAGCTCTCCTTTCAT
**AT3TE72850 F**	CTTCACTCTTTGGGACTGGAAC
**AT3TE72850 R**	TGGTTTCTGAATTGAGGAGGTT
**TSI F**	ATCCAGTCCGAAGAACGCGAACTA
**TSI R**	TCACTTGTGAGTGTTCGTGAGGTC
**AT2TE28020 F**	TCCGCGGTTTAATTGACTAACT
**AT2TE28020 R**	CATAACCCCTAACCCAATTTCA

Primers used for evaluation of mRNA in the T-DNA insertion mutant lines by qPCR.

Primer name	Sequence

**ORC1a 1**	ATGGCTTCTTCTCTGAGTTCCA
**ORC1a 2**	CGATTTGCGATACATTTTAGTTGG
**ORC1a 3**	TCGAAGATTGTCAGATCTGC
**ORC1a 4**	GGTGGCTTAGGAACAACCAC
**ORC1a 5**	ACAATAAGGATCTTCCATGGT
**ORC1a 6**	CAATCTCTCCAAAGTTTTCCT
**ORC1b 7**	AAAAAGTCTGGTCAAAGTCAAACA
**ORC1b 8**	AGCAGCCCATAGATCACCG
**ORC1b 9**	GCATTGAGTGGTCATCGAGTC
**ORC1b 10**	GGCTTGGTAGGCCAATCAAG
**ORC1b 11**	TAAAATCGGTTGCGATCTTGG
**ORC1b 12**	TAATTGGCCAGCCATGGAAG
**ACT2 F**	CCGCTCTTTCTTTCCAAGC
**ACT2 R**	CCGGTACCATTGTCACACAC
