# Supplementary material for: Distinct roles of Arabidopsis ORC1 proteins in DNA replication and heterochromatic H3K27me1 deposition
Source: Nat Commun. 2023 Mar 7;14:1270. doi: 10.1038/s41467-023-37024-8 (PMC9992703; doi:10.1038/s41467-023-37024-8)
Supplement: Supplementary file 3 — Description of Additional Supplementary Files [file 41467_2023_37024_MOESM3_ESM.pdf]

Description of Additional Supplementary Files:

Supplementary Data 1: Raw data of main and supplementary figures

Supplementary Movie 1: Presence of ORC1b during G2 and mitosis

Supplementary Movie 2: Loading of ORC1b during G1 and its degradation at the G1/S transition.
