# Supplementary figures and images for: Distinct roles of Arabidopsis ORC1 proteins in DNA replication and heterochromatic H3K27me1 deposition
Source: Nat Commun. 2023 Mar 7;14:1270. doi: 10.1038/s41467-023-37024-8 (PMC9992703; doi:10.1038/s41467-023-37024-8)

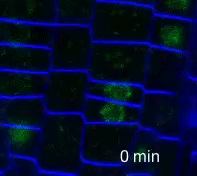

Supplement: Supplementary file 5 — Supplementary Movie 1 [file 41467_2023_37024_MOESM5_ESM.gif]
