# Supplementary material for: Distinct roles of Arabidopsis ORC1 proteins in DNA replication and heterochromatic H3K27me1 deposition
Source: Nat Commun. 2023 Mar 7;14:1270. doi: 10.1038/s41467-023-37024-8 (PMC9992703; doi:10.1038/s41467-023-37024-8)
Supplement: Supplementary file 7 — Reporting Summary [file 41467_2023_37024_MOESM7_ESM.pdf]

nature portfolio

Corresponding author(s): Crisanto Gutierrez

Last updated by author(s): February 19, 2023

Reporting Summary

Nature Portfolio wishes to improve the reproducibility of the work that we publish. This form provides structure for consistency and transparency in reporting. For further information on Nature Portfolio policies, see our Editorial Policies and the Editorial Policy Checklist.

Statistics

For all statistical analyses, confirm that the following items are present in the figure legend, table legend, main text, or Methods section.

n/a	Confirmed	
☐	☒	The exact sample size (*n*) for each experimental group/condition, given as a discrete number and unit of measurement
☐	☒	A statement on whether measurements were taken from distinct samples or whether the same sample was measured repeatedly
☐	☒	The statistical test(s) used AND whether they are one- or two-sided<br>*Only common tests should be described solely by name; describe more complex techniques in the Methods section.*
☒	☐	A description of all covariates tested
☒	☐	A description of any assumptions or corrections, such as tests of normality and adjustment for multiple comparisons
☐	☒	A full description of the statistical parameters including central tendency (e.g. means) or other basic estimates (e.g. regression coefficient) AND variation (e.g. standard deviation) or associated estimates of uncertainty (e.g. confidence intervals)
☒	☐	For null hypothesis testing, the test statistic (e.g. *F*, *t*, *r*) with confidence intervals, effect sizes, degrees of freedom and *P* value noted<br>*Give P values as exact values whenever suitable.*
☒	☐	For Bayesian analysis, information on the choice of priors and Markov chain Monte Carlo settings
☒	☐	For hierarchical and complex designs, identification of the appropriate level for tests and full reporting of outcomes
☒	☐	Estimates of effect sizes (e.g. Cohen's *d*, Pearson's *r*), indicating how they were calculated

Our web collection on statistics for biologists contains articles on many of the points above.

Software and code

Policy information about availability of computer code

Data collection	Flow Cytometry: BD FACS DIVA v8.0.1<br>Confocal Microscopy: Nikon A1R+, Zeiss LSM 710, Zeiss LSM 510 and Zeiss LSM 800<br>Microscopy: Zeiss Axioskop 2 plus Microscope and Leica MZ.9 Binocular Stereo Microscope<br>Western blot: Amersham Imager 600 and Bio-Rad GS-900 Calibrated Densitometer<br>qPCR: Bio-Rad CFX384 Touch Real-Time PCR System
Data analysis	Flow cytometry: FlowJo v10<br>Image analysis: Fiji ImageJ 2.0.0-rc69/1.52i<br>Data analysis: Prism (version 8.2.1), Excel (version 16.16.12) and Sigma Plot (version 14.5)<br>qPCR: Bio-Rad CFX Maestro<br>For Slopes t-test analysis (Suppl Table S1): usablestats.com/calcs/2samplet

For manuscripts utilizing custom algorithms or software that are central to the research but not yet described in published literature, software must be made available to editors and reviewers. We strongly encourage code deposition in a community repository (e.g. GitHub). See the Nature Portfolio guidelines for submitting code & software for further information.

Data

Policy information about availability of data

All manuscripts must include a data availability statement. This statement should provide the following information, where applicable:

- Accession codes, unique identifiers, or web links for publicly available datasets
- A description of any restrictions on data availability
- For clinical datasets or third party data, please ensure that the statement adheres to our policy

Data available on request.

Human research participants

Policy information about studies involving human research participants and Sex and Gender in Research.

Reporting on sex and gender	*Use the terms sex (biological attribute) and gender (shaped by social and cultural circumstances) carefully in order to avoid confusing both terms. Indicate if findings apply to only one sex or gender; describe whether sex and gender were considered in study design whether sex and/or gender was determined based on self-reporting or assigned and methods used. Provide in the source data disaggregated sex and gender data where this information has been collected, and consent has been obtained for sharing of individual-level data; provide overall numbers in this Reporting Summary. Please state if this information has not been collected. Report sex- and gender-based analyses where performed, justify reasons for lack of sex- and gender-based analysis.*
Population characteristics	*Describe the covariate-relevant population characteristics of the human research participants (e.g. age, genotypic information, past and current diagnosis and treatment categories). If you filled out the behavioural & social sciences study design questions and have nothing to add here, write "See above."*
Recruitment	*Describe how participants were recruited. Outline any potential self-selection bias or other biases that may be present and how these are likely to impact results.*
Ethics oversight	*Identify the organization(s) that approved the study protocol.*

Note that full information on the approval of the study protocol must also be provided in the manuscript.

Field-specific reporting

Please select the one below that is the best fit for your research. If you are not sure, read the appropriate sections before making your selection.

☒ Life sciences ☐ Behavioural & social sciences ☐ Ecological, evolutionary & environmental sciences

For a reference copy of the document with all sections, see nature.com/documents/nr-reporting-summary-flat.pdf

Life sciences study design

All studies must disclose on these points even when the disclosure is negative.

Sample size	Attempts to estimate sample size have not been made. For each type of experiment or repeat, different sample sizes were analyzed based on our previous experience (Desvoyes et al., 2020, Nat Plants 6, 1330–1334; Fernández-Marcos et al., 2017, New Phytol 213(1):105-112; Otero et al., 2016, Plant Cell 28: 1361–1371).
Data exclusions	No data were excluded from the analysis.
Replication	All attempts at replicating experiments were successful. In each case, one of them was presented in the figures.
Randomization	Several mutant plants were confirmed using PCR and RT-qPCR, seeds were collected and used randomly for the experimets. Transgenic plants were randomly chosen in the selection plates after transforamtion and confirmed to have fluorescence signal.
Blinding	Blinding was not used in this study. To avoid any bias, we have quantified all samples available in each experiment (e.g., several root epidermal nuclei, as indicated in each figure).

Reporting for specific materials, systems and methods

We require information from authors about some types of materials, experimental systems and methods used in many studies. Here, indicate whether each material, system or method listed is relevant to your study. If you are not sure if a list item applies to your research, read the appropriate section before selecting a response.

Materials & experimental systems

n/a	Involved in the study
☐	☒ Antibodies
☒	☐ Eukaryotic cell lines
☒	☐ Palaeontology and archaeology
☒	☐ Animals and other organisms
☒	☐ Clinical data
☒	☐ Dual use research of concern

Methods

n/a	Involved in the study
☒	☐ ChIP-seq
☐	☒ Flow cytometry
☒	☐ MRI-based neuroimaging

Antibodies

Antibodies used	Anti-H3K27me1 - Millipore (07-488) Lot 3542474<br>Anti-H3K9me2 - Abcam (ab1220) Lot GR212253-5<br>Anti-GFP - ThermoFisher (A-6455) Lot 1853896<br>Anti-BrdU - Becton Dickinson (347580) Lot 57267<br>Anti-GFP - Abcam (ab5450) Lot GR3402450-1<br>Alexa Donkey anti-mouse 555 - ThermoFisher (A31570) Lot 2153958<br>Alexa Donkey anti-rabbit 488 - ThermoFisher (A-21206) Lot 1981155<br>Alexa Goat anti-rabbit 488 - ThermoFisher (A-11034) Lot 48619A<br>Rabbit anti-goat IgG (whole molceule)-Peroxidase - Sigma Aldrich (A5420) Lot 0000161498
Validation	Anti-H3K27me1 and Anti-H3K9me2 antibodies were used previously (Jacob et al., 2009, Nat Struct Mol Biol 16(7): 763–768). Anti-BrdU antibody was used previously (Swanson et al., 2015, Development, 142, 4288-4298). Anti-GFP (ThermoFisher) and Anti-GFP (Abcam) were used previously (Li, et al., 2020, Plant Cell Vol. 32: 3961–3977; Fracchia et al., 2020, Cells 9(10): 2161) respectively.

Flow Cytometry

Plots

Confirm that:

☒ The axis labels state the marker and fluorochrome used (e.g. CD4-FITC).

☒ The axis scales are clearly visible. Include numbers along axes only for bottom left plot of group (a 'group' is an analysis of identical markers).

☒ All plots are contour plots with outliers or pseudocolor plots.

☐ A numerical value for number of cells or percentage (with statistics) is provided.

Methodology

Sample preparation	It is described in Methods. Roots of 7 days old seedlings grown in MS-agar plates or leaves #3/4 of 24 day-old plants grown in soil of the indicated genotypes were chopped with a razor blade in 500 µl of cold Galbraith solution, as described (Desvoyes et al., 2006, Plant Physiol). RNAse at 100 µg/mL and propidium iodide at 50 µg/mL was added before being analyzed in the flow cytometer.
Instrument	FACSCanto A (Becton Dickinson)
Software	BD FACS DIVA v8.0.1<br>FlowJo
Cell population abundance	In all cases 10,000 nuclei were analyzed.
Gating strategy	Two gating criteria were used: propidium iodide (FL2-A)/side scatter area (SSC-A) in a log scale to analyze the ploidy of nuclei and propidium iodide FL3-A/FL3-W in a linear scale to discriminate and exclude nuclei doublets. Representative ploidy profiles are presented in Fig 5.b).

☐ Tick this box to confirm that a figure exemplifying the gating strategy is provided in the Supplementary Information.
