# Supplementary material for: Distinct roles of Arabidopsis ORC1 proteins in DNA replication and heterochromatic H3K27me1 deposition
Source: Nat Commun. 2023 Mar 7;14:1270. doi: 10.1038/s41467-023-37024-8 (PMC9992703; doi:10.1038/s41467-023-37024-8)
Supplement: Supplementary file 2 — Peer Review File [file 41467_2023_37024_MOESM2_ESM.pdf]

nature portfolio

# Peer Review File

## Distinct roles of Arabidopsis ORC1 proteins in DNA replication and heterochromatic H3K27me1 deposition

**Open Access** This file is licensed under a Creative Commons Attribution 4.0 International License, which permits use, sharing, adaptation, distribution and reproduction in any medium or format, as long as you give appropriate credit to the original author(s) and the source, provide a link to the Creative Commons license, and indicate if changes were made. In the cases where the authors are anonymous, such as is the case for the reports of anonymous peer reviewers, author attribution should be to 'Anonymous Referee' followed by a clear attribution to the source work. The images or other third party material in this file are included in the article's Creative Commons license, unless indicated otherwise in a credit line to the material. If material is not included in the article's Creative Commons license and your intended use is not permitted by statutory regulation or exceeds the permitted use, you will need to obtain permission directly from the copyright holder. To view a copy of this license, visit http://creativecommons.org/licenses/by/4.0/.

**Reviewer #1 (Remarks to the Author):**

**Evaluation "Distinct roles of Arabidopsis ORC1 proteins in DNA replication and heterochromatin maintenance"**
**The manuscript presents the investigation of the ORC1 subunit of the replication complex in Arabidopsis, particularly on the role played by the two variants ORC1a, ORC1b. The work combined expression pattern and protein stability analysis using reporter lines, the functional analysis of single mutants and that of one potential complementation line relative to cell cycle progression and root growth, and a histo-immunological analysis of a heterochromatin-associated marker (H3K27me1). The authors conclude for distinct roles for the two variants relative to heterochromatin maintenance.**
**While the results indeed show that both proteins have distinct localization patterns and likely cell cycle dynamics in root nuclei, the differential role in DNA replication and heterochromatin maintenance is not clearly demonstrated. The levels of DNA replication was not assessed, the impact of the slight delay in S-phase progression is not clear; heterochromatin was investigated only using one epigenetic marker, whereas heterochromatin should be also assessed using a common cytological measure (relative heterochromatin fraction, RHF) and maintenance would imply to follow over cell division, or time.**
**In addition, and most importantly, the level of replication of a large number of experiments is either too low or not indicated, which prevents assessing the robustness of the results. Finally, the manuscript in general would benefit from proof-reading by a native speaker to improve clarity and concision.**

**Detailed comments on the lack of replication (or lack of information about it):**
**ORC1a, ORC1b localization analysis is based on the scoring of the number of GFP positive cells per root zone. I understand this is a tedious approach, but n=5 roots is unfortunately too low. It would perhaps be possible to score differently the preferential distribution in the different zones, in a higher number of roots, without scoring individual cells, using the GUS reporters? The strategies to refine the timing of ORC1b synthesis/loading during the cell cycle are pertinent but currently described in a rather confusing way. The following of ORC1b loading (Fig2d) by quantifying the signal in live is very important but only done in 2 cells (also, the panel at 45min show lower signals than at 30min and has been excluded from the plot ? ), also, it is not explained how the cell cycle stage of these cells, qualified as telophase, has been identified. This experiment is critical and very informative and should be strengthened. Similarly, the nice experiment addressing ORC1b degradation at the G1-S transition should be strengthened with additional quantifications and replicates. Same remark for the analysis during endocycles, where "most EdU+" cells should be quantified.**
**Upon treatment with proteasome inhibitors, ORC1b accumulated in "a large fraction of cells", this should be quanrtified. Also , the insets shown in Fig3a for the control and the treated roots do not show the same cell types and absolute levels cannot be compared. This conclusion requires a quantification in bulk for instance using Western Blot, or quantification of GUS activity in root extracts.**
**The experiments showed Figure 4 are in principle very informative and appropriate to address the question, but the lack of information on the level of replication makes it difficult to evaluate the robustness of the results. The number of roots used for experiments in Fig 4a, 4b are not given, there is no error bar on the graph Fig 4b. Replication of this experiment is especially important since the authors use it to conclude that the orc1b mutant shows a delay in S-phase progression. The complementation experiment is not clear. The authors claim that introducing ORC2-GFP partially complements the sensitivity to the drug but it is left to the reader to compare the curve and numbers on the upper right two graphs in Fig4c. A rough estimation gives a root length of ca 18um in orc1b-Aphi versus 22 (?)um in orc1b complemented with ORC1b-GFP and treated with Aphi? For validating the conclusion, comparing the root length for a specific time point with the relevant genotypes , replicates, and statistical tests is necessary. The interpretation by the author excludes a defect in the DNA damage response by measuring the transcript levels of RAD51, BRCA1 and PARP1.**

**Unfortunately, there is no indication of biological replication of this experiment shown as supplementary Figure S6.**
**The experiment to measure the levels of H3K27me1 and H3K9me2 (Fig 5 and supplementary Fig S7) is indeed essential to understanding the differential role of ORC1a and ORC1b in heterochromatin maintenance. The approach chosen by the authors is immunostaining. Yet, comparing the absolute fluorescence intensities in the wild-type and the mutant as shown Fig 5a-b is equivalent to compare the levels of an RT-PCR amplification without normalization against an endogenous, stably expressed control. Fluorescence signals can potentially vary between samples due to differences in antibody penetration, variable preservation of the epitope during fixation, different depth of the root in the preparation (i.e. relative to the coverslip and objective collecting light) or a combination of all. In addition, fluorescence levels are influenced by the antibody reaction time and temperature. It is thus essential to normalize the signals with another chromatin component (e.g. H3) and to perform measurements on different biological samples, prepared independently. This might have been done here, but it is not indicated. In addition, different results are expected for nuclei at the meristem, elongation or differentiation zones which have dramatically distinct heterochromatin content and endoreplication levels. Since these aspects are directly related to an ORC function, this should be carefully considered, measured and discussed. The plots shown Fig 5c are potentially very illustrative yet arise from single nuclei and are thus misleading – such plots would need to be done on several nuclei, from independent biological replicates to convey a robust information. Particularly, the choice of the lines across the nuclei, chosen to plot the absolute signals do not capture equally well chromocenters in the wild-type and mutants. Hence it is difficult to compare and draw conclusions.**

**Additional issues relative to the text or figures**
**Page 4: "Globally, a very large percentage of cells in the root were devoid of ORC1a-GFP (92.7% in the proliferation zone (PZ) and 36.5% in the transition and elongation zones": please add " show no signal").**
**Page 4: The explanation of the experiment with brdU and EdU colabeling requires reformulation, currently confusing. Possibly, the full details is only shown and explained in the supplements whereas the main text could simply mention " A dual color pulse-chase experiment enabling the identification of G2 cells confirmed ORC1b expression at G2, similar to the human Orc1" ?**
**Page 5 "expressing the G2 marker CYCB1;1-GFP" : isn't this marker expressed at the G2-M transition? Please specify as it is important for the question posed "to define when ORC1b is synthetized in G2", and the aim is to refine the beginning vs the end of G2 (?). Also clarify the next sentence "..is loaded in G2": end or beginning? (if this is the question). Also, give a reference for the timing of this protein accumulation in Arabidopsis.**
**Page 5 "in cells that just finished telophase was 15-20%", to which dataset does this number range correspond? How many cells were analysed? ; follow up sentence is not clear "further loading in G1", how is this concluded from Fig2d is not clear, and why "further loading" since G1 occurs before G2?**
**Page 5 "most EdU+ endoreplicating cells" should read "..EdU-positive, endoreplicating cells"**
**Page 5"incubation with …MG132 and epoxomicin..", specify that these compounds are proteasome inhibitors.**
**Page 5: The concept behind the toxicological experiments targeting the proteasome-dependent protein degradation system should be briefly described for didactic purposes. Why MG132 and epoxomicin are mixed?**
**Page 5: "The differences in expression domains, subcellular localization.." , the proteins do not have different subcellular localization, they are both nuclear. They have different sub-nuclear distribution, or, more precisely, the dynamics of their sub-nuclear distribution is different (as they can locate in both eu and heterochromatin but likely at different time point in the cell cycle)**
**Page 6: "showed a trend to be slightly larger although the differences did not have statistical significance" , what should we conclude from this statement? If this is**

**statistically not different, it means that the sample size and variation of measurements do not allow to conclude about a difference, hence the message should be "no significant differences in meristem length and number of cortical cells". If the authors believe that the mutant have a phenotype in this respect, they should perform additional measurements (currently, n=12 roots only for 4 dap old seedlings).**

**Figure 1**
**-panel h and i: the legends 'OCR1a/b expression domains' are too small. It would be easier to have a legend on top 'ORC1a-GFP', 'ORC1b-GFP' as for panels a-b, and eventually a box encompassing the expression domain.**
**-panel i: the line drawing the expression domain of ORC1b-GFP is larger than the observed GFP domain, which is stronger towards the tip as is the expression domain of ORC1b-GUS. Please adjust or explain**
**- graphs panel h and i: the category "empty" should be renamed for "no signal"**
**Figure 4**
**-panel a, please provide here on in supplement typical EdU labeling that allowed the quantification as early, mid or late S-phase**
**Supplementary Fig S1:**
**- please give the total n=nuclei scored**
**- Does the % of GFP cells represents the number of GFP positive cells in one cell type category per root, or the number of GFP positive cell of one specific cell type relative to (as a fraction of) all other cell types?**
**- how is the s.e.m calculated? Does it represent the variation between roots (for each fraction of cell type) ? this does not appear a valid approach for QC with only 2 cells – see next point**
**- for n=8 roots, the number of nuclei scored in the QC are expected to be 16 (?). I am not sure it is valid to express a % of GFP expressing cells for this sample size. 10% average occurrence means 0.2 cells per root. It may be better to express the frequency of roots showing GFP in each cell type?**
**Figure 5: which root cell types were measured? Imaging in the epidermis vs cortex is expected to provide different intensity levels due to different depth ; also nuclei at the meristem, elongation and differentiation zones have distinct heterochromatin content and endoreplication levels hence will influence the measurements. In addition to normalization (see comment above), biological replication (see comment above), information on the root tissue and zone is relevant. Panel c shows single nuclei, how representative is this? Also, not all nuclei show clear chromocenters (DAPI curve; images are too small to visualize)**
**Supplementary Fig S2: the scheme and legend are confusing. It may be easier to show 3 cases only presented along a linear, time progression, and to comment them one by one. Each panel would then represent one case, with a x axis being time and cell cycle stages, arrows showing the BrdU, cold (T) and EdU pulses. Case 1, could be "cell 2" leading to a cell labeled with EdU only, Case 2 could be "cell 4" leading to a cell labelled with BrdU only, Case 3 as "cell 8" leading to co-labeling of BrdU and EdU and Case 4 as cell 1 (or 7) leading to no labeling.**

**Supplementary Fig S3: Which primers were used in the RT- control reaction?**
**Methods: antibody incubation time and temperature missing – those influence dramatically the fluorescence levels.**

**Reviewer #2 (Remarks to the Author):**

**Review Vergara et al.**

**This manuscript by Vergara et al. describes a characterisation of ORC1 function in Arabidopsis thaliana. ORC1 is a key component of the conserved Origin Recognition**

**Complex (ORC) whose main role lies in driving the assembly of the replication machinery to origin or replication, and as such is key in driving DNA replication. The Arabidopsis genome encodes two ORC1 genes (ORC1a, and ORC1b) and the authors use a variety of approaches to show that of these 2 gene, Orc1b behaves more like a classical ORC component, both in terms of expression profile and in terms of phenotype. Orc1a, on the other hand, is suggested by the authors to play a specialized role in chromocenters/heterochromatinized regions. The authors thus speculate that in arabidopsis, the two ORC1 genes have undergone specific functionalization, as has been seen for ORC1 gene paralogs in other species, most notably Orc1 and Sir3 in budding yeast.**

**In principle, the observations of the authors give some tantalizing glimpses into the potential roles for these two, highly identical but also unique, gene paralogs. However, the manuscript currently falls short in terms functional insight. I encourage the authors to focus on gaining more insight into the potential role of Orc1a in heterochromatin formation and/or maintenance. If they can add more insight into to this potentially interesting finding, this would improve the work greatly.**

**Specific points:**

**-It would help the reader to get a better idea of the difference/similarities if the authors provide an in-depth analysis of the protein sequences of Orc1a and Orc1b, also in relation to other ORC1 genes in other species. Where are the differences, and are these differences concentrated in specific domains? Is the BAH domain, and AAA+ comparable? Are any amino acid changes expected to alter protein function, based on prior knowledge?**
**-The current connection between Orc1a and heterochromatin is not developed enough. Can the authors add more insight here?**
**-In general, can the two alleles that are used (orc1a-2 and orc1b-2) be compared vis-a-vis penetrance? What are the mutations that generated these alleles? Can it be said that are they equally affecting protein function? If this cannot be tested rigorously, it remains challenging to make strong conclusions regarding specific functions in which one of the genes is 'more important' than the other. In relation to this: can a double mutant be made (i.e. orc1a-2, orc1b-2)? If so, what is the phenotype?**
**-To what extent does the fact that many other DNA replication genes show duplications (the authors mention Cdc6 and Cdt1) influence potential interpretations towards functionalization? In the example that is used (budding yeast), this does not appear to be the case, and Orc1 stands out as having a diverged paralog (Sir3). How could this be interpreted?**
**-Page 3, first paragraph of results: '..orc1b expression co-localizes…'. Strange wording, please rephrase.**
**-Page 5, line 3: '…confirming that Orc1b was loaded in G2..'. Loaded seems to be potentially confusing terms here: thes experiments only reveal localization patterns, whereas loading, in the context of ORC, might be interpreted to mean something more specific. Please rephrase.**
**-Page 5, third paragraph: ' live imaging…'. These findings do to reinforce that Orc1 is degraded per se. Please rephrase.**
**-Page 5, fourth paragraph: these experiments suggest that proteosomal degradation might influence Orc1 protein levels, but do not 'establish' a direct link. The effect can conceivably be rather indirect. Rephrase statements.**
**-Page 6, first paragraph: what is the conclusion of these experiments?**
**-Page 6, second paragraph: the section describing the BrdU/EdU pulses is hard to digest in its current form, please rewrite. In terms of experiments: what would the effect of a classical, strong S-phase mutant be in such an analysis, for reference? Can such a mutant be tested?**
**-Page 7, first paragraph: it would be very interesting if the authors can add more insight into this part. Is heterochromatin impaired during all cell cycle phases? Is it different in G1 vs S/G2? What is the rationale about linking orc1a to atxr5/6? Is there a direct**

**functional connection? Related: in Figure 5: in Figure 5B, please include in the same analysis the single mutants (and the orc1-a without Orc1a-GFP).**

**Reviewer #3 (Remarks to the Author):**

**This study presents a comprehensive overview of the expression/localization dynamics of the ORC1A and ORC1B proteins in plant tissues, cells and subnuclear compartments, from which it is derived that they have different functions. ORC1B plays a role in DNA replication in defining the origin of replications, while the role of ORC1A has diverged and appears to be important for the maintenance of the repressive histone mark H3K27me1. Therefore, this work provides the first detailed characterization in plants of the different functions of these proteins, and as such, it represents an important contribution to our understanding of DNA replication in eukaryotes. The conclusions presented are justified, and the quality of the work is very good.**

**Please see below for comments that may improve the manuscript.**

**1. Characterization of the single orc1A and orc1b mutants clearly validates that these proteins have some independent functions in plants. However, it would have been interesting to create a double mutant to see if these proteins play partially overlapping roles in some instances. Creating such a double mutant in Arabidopsis and re-doing some of the analyses would take many months, so I don't think doing this work is a requirement for publication. However, if the double mutant is available, some basic characterizations of its phenotype(s) could be presented to answer the question of partial overlap.**

**2. The effect of the orc1a mutant on lowering H3K27me1 levels is interesting, and could benefit from being characterized a little bit further. For example, atxr5 atxr6 double mutants show re-replication phenotypes on FACS profiles. Do we see the same phenotypes for the orc1a mutant? What about transcriptional derepression in heterochromatin for the orc1a mutant? If similar to the atxr5 atxr6 mutant, then we could expect some transposable elements to be reactivated in the orc1a mutants?**

**Minor edits:**

**1. Fig. 1H and 1I: The name used for the upper part of the root in the Figure is EZ+DZ. However, the name for this zone used in the text is TZ+EZ.**

**2. I would slightly alter the following sentence to clarify the meaning, assuming the edits do not change the meaning of the sentence:**

**"The nuclear pattern of ORC1b-GFP was completely different, with a preferential localization in the proliferation zone (50.1%) and in the transition and elongation zone (51.5%; Fig. 1i)."**

**to this sentence:**

**"The root-specific pattern of ORC1b-GFP localization was completely different compared with ORC1A-GFP, as shown by a majority cells containing ORC1B in both the proliferation zone (50.1%) and in the transition and elongation zone (51.5%; Fig. 1i)"**

**3. "The patchy pattern of ORC1b protein in proliferating cells supports a cell cycle regulation."**

**I would change to:**

**"The patchy pattern of ORC1b in proliferating cells suggests that expression of the protein is regulated during the cell cycle."**

**4. Fig. 1 is missing labels for panels E, F, and G**

**5. Legend of Fig. 1. An arrowhead for the mature guard cells is mentioned in the legend, but omitted on the figure. It's also hard to distinguish the solid color line indicating the mean and SEM (?) on panels H and I. Maybe changing the color of the line to black would help. Finally, it says at the bottom of the legend that the most common patterns in panels H and I are shown in bold. Since green and blue colors are used, I would refer to the colors instead.**

**6. Fig. 2. In panel C, I am not sure what the star indicates (localization marker?), and there is no mention of it in the legend. There is also a typo in the middle of the legend, with "Th" missing an "e".**

**7. Supplementary Fig. 3. Typically, for these T-DNA validation experiments, primer pairs will also be used to assess expression upstream and/or downstream of the T-DNA expression, to see if a partial transcript is still expressed. This is particularly important if the T-DNA is located in the promoter or near the 3'end of a gene, which is not the case here. However, it is still informative to see the impact of T-DNA on the expression of any part of a gene.**

**Also, it's not clear on panel A what part of the gene is shown. Does it go from the start codon to the stop codon, with the 3'UTR indicated by a white box at the end of the genes? Also, it would be good to remind the readers in the legend that the ORC1A and ORC1B genes do not have introns, thus confirming that the T-DNA insertions are in exons.**

**8. Supplementary Fig. S6. This figure is missing SEM and statistics.**

**9. Supplementary Fig. S7. It would be informative to include the correlation coefficients on the panels**

**Reviewer #1 (Remarks to the Author):**

Evaluation "Distinct roles of Arabidopsis ORC1 proteins in DNA replication and heterochromatin maintenance". The manuscript presents the investigation of the ORC1 subunit of the replication complex in Arabidopsis, particularly on the role played by the two variants ORC1a, ORC1b. The work combined expression pattern and protein stability analysis using reporter lines, the functional analysis of single mutants and that of one potential complementation line relative to cell cycle progression and root growth, and a histo-immunological analysis of a heterochromatin-associated marker (H3K27me1). The authors conclude for distinct roles for the two variants relative to heterochromatin maintenance. While the results indeed show that both proteins have distinct localization patterns and likely cell cycle dynamics in root nuclei, the differential role in DNA replication and heterochromatin maintenance is not clearly demonstrated. The levels of DNA replication was not assessed, the impact of the slight delay in S-phase progression is not clear; heterochromatin was investigated only using one epigenetic marker, whereas heterochromatin should be also assessed using a common cytological measure (relative heterochromatin fraction, RHF) and maintenance would imply to follow over cell division, or time.

**R: Initial observations revealed that the amount of heterochromatin did not change dramatically in the orc1a-2 mutant. Therefore, we focused on identifying possible defects at the histone modification level. The, we found clear defects in H3K27me1 deposition, whereas the H3K9me2 signal remained as in the wild type.**

In addition, and most importantly, the level of replication of a large number of experiments is either too low or not indicated, which prevents assessing the robustness of the results. Finally, the manuscript in general would benefit from proof-reading by a native speaker to improve clarity and concision.
**R: We appreciate the detailed review provided. We have tried to address all the questions, in most cases this has been possible, while in others we provide arguments in support of our view.**

Detailed comments on the lack of replication (or lack of information about it):
ORC1a, ORC1b localization analysis is based on the scoring of the number of GFP positive cells per root zone. I understand this is a tedious approach, but n=5 roots is unfortunately too low. It would perhaps be possible to score differently the preferential distribution in the different zones, in a higher number of roots, without scoring individual cells, using the GUS reporters?
**R: The suggested use of a GUS reporter (transcriptional?) is not appropriate here since we are interested in studying the dynamics at the protein level, where the availability of ORC1b is regulated. Also, the resolution will never be comparable. In case that the reviewer refers to a GUS translational fusion, we will probably face similar problems. Therefore, we have increased the number of roots scored, as indicated. In any case, we consider that the total number of nuclei scored (now ~1000 per experiment) is sufficiently high as to be representative of the whole population. We have included the new data in the revised Fig 1i and j). Below we show for your information a comparison of the results shown in the original manuscript and the new data (new Fig. 1i and j), which are**

indistinguishable. It is important to keep in mind that these distributions do not refer to intensity of the GFP signal but rather we scored not only the labeling pattern in each case but also the positional information within the root, which is a crucial aspect of our study. This is the reason why we prefer to maintain this strategy to avoid loss of information.**

<u>PREVIOUS</u> <u>NEW</u>

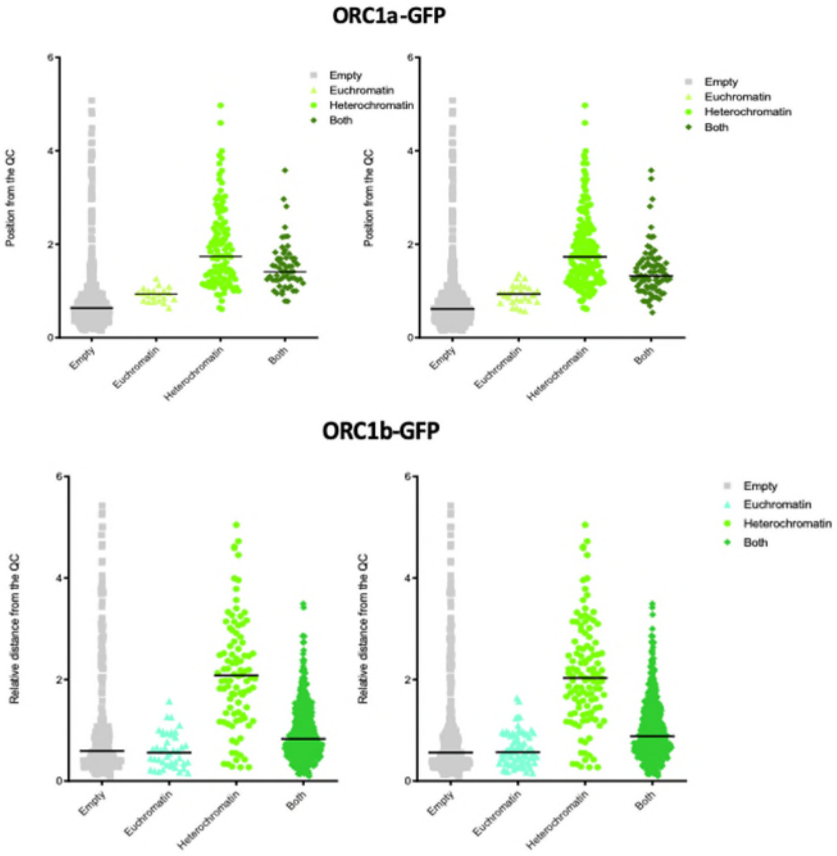


**Other panels have been also revised according to your and other reviewers' comment.**

The strategies to refine the timing of ORC1b synthesis/loading during the cell cycle are pertinent but currently described in a rather confusing way. The following of ORC1b loading (Fig2d) by quantifying the signal in live is very important but only done in 2 cells (also, the panel at 45min show lower signals than at 30min and has been excluded from the plot ? ), also, it is not explained how the cell cycle stage of these cells, qualified as telophase, has been identified. This experiment is critical and very informative and should be strengthened.
**R: We have rewritten this description trying to make it more clear. We have also made new quantifications on live-imaging experiments. The left panels in the new Fig. 2d and f contain representative examples. Regarding the loading, quantifications were done in pairs of nuclei easily identified in mitosis, telophase in most cases (to be sure that we are measuring accumulation during the next G1). For the degradation, nuclei were chosen based on their high GFP signal and then followed during the indicated times. We have included all the data points in each time.**

Similarly, the nice experiment addressing ORC1b
degradation at the G1-S transition should be strengthened with additional quantifications and replicates.

**R: See response above. We have quantified the GFP and EdU signals in these experiments where can be clearly seen that the two signals are excluded (new graph in Fig 2e) in >95% of cells (n=137 cells). This analysis corresponds to nuclei in the RAM.**

Same remark for the analysis during endocycles, where "most EdU+" cells should be quantified.
**R: We have quantified the GFP and EdU signals in these experiments where can be clearly seen that the two signals are excluded (new graph in Fig 2g) in >95% of cells. This analysis corresponds to nuclei in the endoreplication region.**

Upon treatment with proteasome inhibitors, ORC1b accumulated in "a large fraction of cells", this should be quanrtified. Also , the insets shown in Fig3a for the control and the treated roots do not show the same cell types and absolute levels cannot be compared. This conclusion requires a quantification in bulk for instance using Western Blot, or quantification of GUS activity in root extracts.
**R: We have revised the nuclei included in the insets. In panel a, we focused on the region just above the RAM because this is where most ORC1a expression is detected. In panel b we show two regions. In all cases we have considered the epidermal layer and within it the cell files that appear in the center of the root, as indicated.**
**Regarding alternative ways to detect protein accumulation, we have included an evaluation of protein amount after treatment with proteasome inhibitors by Western blot. We have now used MLN4924 and bortezomib (instead of MG132 and epoxomicin), the latter also evaluated by confocal microscopy. The WB has been also quantified, as requested (new Fig. 3c). Note that based on the expression pattern of ORC1b, restricted largely to G1 cells, the accumulation after treatment with proteasome inhibitors is relatively small.**

The experiments showed Figure 4 are in principle very informative and appropriate to address the question, but the lack of information on the level of replication makes it difficult to evaluate the robustness of the results. The number of roots used for experiments in Fig 4a, 4b are not given, there is no error bar on the graph Fig 4b. Replication of this experiment is especially important since the authors use it to conclude that the orc1b mutant shows a delay in S-phase progression.
**R: We have now expanded the analysis (new Fig. 4b).**

The complementation experiment is not clear. The authors claim that introducing ORC2-GFP partially complements the sensitivity to the drug but it is left to the reader to compare the curve and numbers on the upper right two graphs in Fig4c. A rough estimation gives a root length of ca 18um in orc1b-Aphi versus 22 (?)um in orc1b complemented with ORC1b-GFP and treated with Aphi? For validating the conclusion, comparing the root length for a specific time point with the relevant genotypes , replicates, and statistical tests is necessary.
**R: We are not fully sure about this comment. We have used ORC1 (ORC2-GFP must be a typo) and the root length is measured in mm. We have compared the root length in the different genotypes after treatment with aphidicolin, and provided the conclusion. A summary of the results of comparing the values at the end of the experiment is now shown in a separate panel (Fig. 4d), as requested, indicating the statistical significance of the different values.**

The interpretation by the author excludes a defect in the DNA damage response by measuring the transcript levels of RAD51, BRCA1 and PARP1. Unfortunately, there is no indication of biological replication of this experiment shown as supplementary Figure S6.
**R: We do no find a valid rationale to justify a defect in the response of the genes measured to the aphidicolin treatment, and this is the reason for not pursuing this line. In any case, as requested by the reviewer, we have done the corresponding analysis with three biological replicates and 2 technical replicates for each gene. The results are now included as the new Supplementary Fig. 5.**

The experiment to measure the levels of H3K27me1 and H3K9me2 (Fig 5 and supplementary Fig S7) is indeed essential to understanding the differential role of ORC1a and ORC1b in heterochromatin maintenance. The approach chosen by the authors is immunostaining. Yet, comparing the absolute fluorescence intensities in the wild-type and the mutant as shown Fig 5a-b is equivalent to compare the levels of an RT-PCR amplification without normalization against an endogenous, stably expressed control. Fluorescence signals can potentially vary between samples due to differences in antibody penetration, variable preservation of the epitope during fixation, different depth of the root in the preparation (i.e. relative to the coverslip and objective collecting light) or a combination of all. In addition, fluorescence levels are influenced by the antibody reaction time and temperature. It is thus essential to normalize the signals with another chromatin component (e.g. H3) and to perform measurements on different biological samples, prepared independently. This might have been done here, but it is not indicated.
**R: Sorry, but we are not fully sure to share your opinion. In the example that you use of an RT-PCR experiment is not comparable to use different antibodies to detect two different marks by immunostaining. This is the reason to use an internal control, the signal of H3K9me2, measured in the same nuclei on those we measured the H3K27me1 signal. We might have made the average H3K27me1 values relative to the average H3K9me2 values (which did not vary among the genotypes used), or divide them for each individual nucleus. Therefore, we are convinced that showing all values for each nucleus and having the view of the entire population of nuclei is much more informative than making any normalization, losing the information of individual marks. Here, we have analyzed the regression of the cloud of data, as specified in the revised version (new Supplementary Fig. 6 and Supplementary Table 1). In addition, we have used as a positive control the atxr5,atxr6 double mutant, known to have defects in H3K27me1 deposition (but not in H3K9me2 levels), which showed a phenotype indistinguishable from the orc1a-2 mutant in the H3K27me1 mark level. Technical details of the immunocytological experiments are now added in Methods.**

In addition, different results are expected for nuclei at the meristem, elongation or differentiation zones which have dramatically distinct heterochromatin content and endoreplication levels. Since these aspects are directly related to an ORC function, this should be carefully considered, measured and discussed.
**R: You are right. Data shown in Fig. 5a and Supplementary Fig. 6 were collected in the region of the root where ORC1a is expressed, according to results of Fig. 1a.**

The plots shown Fig 5c are potentially very illustrative yet arise from single nuclei and are thus misleading – such plots would need to be done on several nuclei, from independent biological replicates to convey a robust information. Particularly, the choice of the lines across the nuclei, chosen to plot the absolute signals do not capture equally well chromocenters in the wild-type and mutants. Hence it is difficult to compare and draw conclusions.
**R: Again, these plots were shown simply to illustrate representative results obtained with hundreds of nuclei. Lines were arbitrarily set to cover a few chromocenters, as indicated. We understand that possibly they are not very informative and redundant with the rest of data provided. Also, the question is how many of these nuclear tracks would be necessary to show. Therefore, we have decided to delete this from the revised version.**

Additional issues relative to the text or figures
Page 4: "Globally, a very large percentage of cells in the root
were devoid of ORC1a-GFP (92.7% in the proliferation zone (PZ) and 36.5% in the transition and elongation zones": please add " show no signal").
**R: Done.**

Page 4: The explanation of the experiment with brdU and EdU colabeling requires reformulation, currently confusing. Possibly, the full details is only shown and explained in the supplements whereas the main text could simply mention " A dual color pulse-chase experiment enabling the identification of G2 cells confirmed ORC1b expression at G2, similar to the human Orc1" ?
**R: Thank you. We have rephrased this idea, including your suggestion: "This dual color pulse-chase experiment, enabling the identification of G2 cells (those BrdU positive), confirmed that ORC1b-GFP protein starts to accumulate in G2 (Fig. 2b), similar to the human Orc1 protein".**

Page 5 "expressing the G2 marker CYCB1;1-GFP" : isn't this marker expressed at the G2-M transition? Please specify as it is important for the question posed "to define when ORC1b is synthetized in G2", and the aim is to refine the beginning vs the end of G2 (?).
**R: To be strict, it is a marker of late G2, prophase and metaphase, since it is rapidly degraded in anaphase. Corrected, as requested.**

Also clarify the next sentence "..is loaded in G2": end or beginning? (if this is the question). Also, give a reference for the timing of this protein accumulation in Arabidopsis.
**R: We have included "indicating that ORC1b starts to accumulate in late G2". We have also included two references for the timing of CYCB1;1 accumulation (Shaul et al., 1996; Colon-Carmona et al., 1999).**

Page 5 "in cells that just finished telophase was 15-20%", to which dataset does this number range correspond? How many cells were analysed? ;
**R: Comparing the signal in G2 and in late G1, just prior to degradation at the G1/S transition. This result is derived from data shown in several experiments shown in Fig. 2.**

follow up sentence is not clear "further loading in G1", how is this concluded from Fig2d is not clear, and why "further loading" since G1 occurs before G2?

**R: Since ORC1b starts to accumulate in G2 and remains during mitosis, we analyzed its dynamics in the G1 phase (next cycle) of the daughter cells, where it is observed that the amount of ORC1 increases to a maximum in G1. This is the reason to use the term "further loading in G1" (after an initial loading in the G2 of the previous cell cycle).**

Page 5 "most EdU+ endoreplicating cells" should read "..EdU-positive, endoreplicating cells"
**R: Corrected.**

Page 5"incubation with …MG132 and epoxomicin..", specify that these compounds are proteasome inhibitors.
**R: Note that we have deleted the experiments with MG132 and epoxomicin, and included new data with bortezomib.**

Page 5: The concept behind the toxicological experiments targeting the proteasome-dependent protein degradation system should be briefly described for didactic purposes. Why MG132 and epoxomicin are mixed?
**R: Please, see previous point.**

Page 5: "The differences in expression domains, subcellular localization.." , the proteins do not have different subcellular localization, they are both nuclear. They have different sub-nuclear distribution, or, more precisely, the dynamics of their sub-nuclear distribution is different (as they can locate in both eu and heterochromatin but likely at different time point in the cell cycle)
**R: Thank you. This sentence has been rewritten, as suggested: "The differences in expression domains of ORC1a and ORC1b, in the dynamics of their subnuclear distribution during the cell cycle and in their stability strongly suggested that they play distinct roles.**

Page 6: "showed a trend to be slightly larger although the differences did not have statistical significance" , what should we conclude from this statement? If this is statistically not different, it means that the sample size and variation of measurements do not allow to conclude about a difference, hence the message should be "no significant differences in meristem length and number of cortical cells". If the authors believe that the mutant have a phenotype in this respect, they should perform additional measurements (currently, n=12 roots only for 4 dap old seedlings).
**R: The sentence has been changed, as requested: "We found that the size of root meristem cortical cells and the number of cortical cells were statistically undistinguishable in wild type, orc1a-2 and orc1b-2 roots."**

Figure 1
-panel h and i: the legends 'OCR1a/b expression domains' are too small. It would be easier to have a legend on top 'ORC1a-GFP', 'ORC1b-GFP' as for panels a-b, and eventually a box encompassing the expression domain.
-panel i: the line drawing the expression domain of ORC1b-GFP is larger than the observed GFP domain, which is stronger towards the tip as is the expression domain of ORC1b-GUS. Please adjust or explain
- graphs panel h and i: the category "empty" should be renamed for "no signal"
**R: These changes have been introduced in the revised Fig. 1.**

Figure 4
-panel a, please provide here on in supplement typical EdU labeling that allowed the quantification as early, mid or late S-phase
**R: This has been introduced in the revised Fig. 4a.**

Supplementary Fig S1:
- please give the total n=nuclei scored
- Does the % of GFP cells represents the number of GFP positive cells in one cell type category per root, or the number of GFP positive cell of one specific cell type relative to (as a fraction of) all other cell types?
- how is the s.e.m calculated? Does it represent the variation between roots (for each fraction of cell type) ? this does not appear a valid approach for QC with only 2 cells – see next point
- for n=8 roots, the number of nuclei scored in the QC are expected to be 16 (?). I am not sure it is valid to express a % of GFP expressing cells for this sample size. 10% average occurrence means 0.2 cells per root. It may be better to express the frequency of roots showing GFP in each cell type?

**R: Since the information obtained is not actually very relevant to the core of the manuscript we decided to remove this analysis.**

Figure 5: which root cell types were measured? Imaging in the epidermis vs cortex is expected to provide different intensity levels due to different depth ; also nuclei at the meristem, elongation and differentiation zones have distinct heterochromatin content and endoreplication levels hence will influence the measurements. In addition to normalization (see comment above), biological replication (see comment above), information on the root tissue and zone is relevant.
**R: All measurements have been carried in the epidermis, precisely to avoid possible problems, as you indicate. This is made explicit. Since ORC1a is not detected in the meristem, we restricted our measurements of the histone marks of nuclei found in the region where ORC1a is detected, as we indicate elsewhere. Thus, scoring was restricted to a region covering from 0.7 of the RAM to the shootward region of the root, as defined in Fig. 1h and 1i (position 0 corresponds to the QC and position 1 to the RAM boundary. As justified earlier, the H3K27me1 signal was compared to the H3K9me2 signal, which does not change in any of the genotypes used. Given the variability of individual signal intensities, normalization with either DAPI of H3K9me2 values does not work in providing a better information. Consequently, we have compared the whole cloud by regression analysis, as shown in Supplementary Fig. 6.**

Panel c shows single nuclei, how representative is this? Also, not all nuclei show clear chromocenters (DAPI curve; images are too small to visualize)
**R: Again, these plots were shown simply to illustrate representative results obtained with hundreds of nuclei. Lines were arbitrarily set to cover a couple of chromocenters, as indicated. We understand that possibly they are not very informative. Also, the question is how many of these nuclear tracks would be necessary to show. Therefore, we have decided to delete this from the revised version.**

Supplementary Fig S2: the scheme and legend are confusing. It may be easier to show 3 cases only presented along a linear, time progression, and to comment them one by one. Each panel would then represent one case, with a x axis being time and cell cycle stages, arrows showing the BrdU, cold (T) and EdU pulses. Case 1, could be “cell 2” leading to a cell labeled with EdU only, Case 2 could be “cell 4” leading to a cell labelled with BrdU only, Case 3 as “cell 8” leading to co-labeling of BrdU and EdU and Case 4 as cell 1 (or 7) leading to no labeling.
**R: We have redesigned the scheme, incorporating your suggestion, to simplify it, and also provided a more detailed explanation in the text. We highly prefer to maintain several cells with different behavior to illustrate the situation of an asynchronous cell population, as it is the case in the RAM.**

Supplementary Fig S3: Which primers were used in the RT- control reaction?
**R: A new Suppl. Fig. has been included addressing not only your request but also that of another reviewer. It now includes data using primer pairs, as indicated. Primers are listed in Supplementary Table 2**

Methods: antibody incubation time and temperature missing – those influence dramatically the fluorescence levels.
**R: This has been specified in the revised version of Methods: “Samples were incubated with the primary antibodies overnight at 4$^{o}$C and with secondary antibody for 1h at room temperature.”**

**Reviewer #2 (Remarks to the Author):**

Review Vergara et al.

This manuscript by Vergara et al. describes a characterisation of ORC1 function in Arabidopsis thaliana. ORC1 is a key component of the conserved Origin Recognition Complex (ORC) whose main role lies in driving the assembly of the replication machinery to origin or replication, and as such is key in driving DNA replication. The Arabidopsis genome encodes two ORC1 genes (ORC1a, and ORC1b) and the authors use a variety of approaches to show that of these 2 gene, Orc1b behaves more like a classical ORC component, both in terms of expression profile and in terms of phenotype. Orc1a, on the other hand, is suggested by the authors to play a specialized role in chromocenters/heterochromatinized regions. The authors thus speculate that in arabidopsis, the two ORC1 genes have undergone specific functionalization, as has been seen for ORC1 gene paralogs in other species, most notably Orc1 and Sir3 in budding yeast.
In principle, the observations of the authors give some tantalizing glimpses into the potential roles for these two, highly identical but also unique, gene paralogs. However, the manuscript currently falls short in terms functional insight. I encourage the authors to focus on gaining more insight into the potential role of Orc1a in heterochromatin formation and/or maintenance. If they can add more insight into to this potentially interesting finding, this would improve the work greatly.
**R: We can assure you that this is something that we have tried hard, but some of the lines revealed more time-consuming and complex than initially anticipated (see below). We have included in this manuscript all the confirmed information available. More detailed studies should follow in the line indicated, but considered now out of the scope of this initial set of data highlighting the different behavior of the two ORC1 proteins. We do hope that you agree.**

Specific points:

-It would help the reader to get a better idea of the difference/similarities if the authors provide an in-depth analysis of the protein sequences of Orc1a and Orc1b, also in relation to other ORC1 genes in other species. Where are the differences, and are these differences concentrated in specific domains? Is the BAH domain, and AAA+ comparable? Are any amino acid changes expected to alter protein function, based on prior knowledge?
**R: We have published (Ocaña-Pallarès et al., 2020) a detailed phyllogenetic study of ORC1 proteins, their domains and the origin of duplications. Following your request, we have now included a short description of the two Arabidopsis proteins, referring to the published paper for ORC1 of other species, and highlighting their differences. As far as we can tell, the relevance of the differences observed is not known, based on prior knowledge, and should be subject of future studies. A new panel has been included in Fig. 1 providing a summary of the major ORC1 domains and comparing Arabidopsis with S. cerevisiae and human Orc1 proteins.**

-The current connection between Orc1a and heterochromatin is not developed enough. Can the authors add more insight here?

**R: With the current data, this would remain speculative. We have expanded some of the sentences in the discussion, based on your comment and that of another reviewer (a comment on the possible re-replication phenotype and TE activation, see also below). We have done new flow cytometry experiments to address this point. Neither the orc1a-2 nor the atxr5/6 mutant show a clear re-replication phenotype in nuclei isolated from roots (new Fig 5b). The re-replication phenotype of the atxr5/6 double mutant is detected in 4-week leaves (Jacob et al., 2010). Thus, we also looked at the flow cytometry profiles in 4 week-old leaves and confirmed that the atxr5/6 mutant shows the typical phenotype but that the orc1a-2 seems to be wild type. This is consistent with the absence of any detectable signal of ORC1a-GFP in leaves (young or old leaves). Therefore, it seems that the loss of ORC1 or ATXR5/6 proteins does not lead to a re-replication phenotype in roots, consistent with a recent report (Potok et al., 2022, doi:** 10.1073/pnas.2115570119 **). It is important to keep in mind that the ploidy level reached in roots is not as high as in old leaves. We have included this analysis in a new Fig. 5b and a new Suppl. Fig. 7, its description in the Results and a short comment in the Discussion.**
**Regarding the TE derepression observed in the atxr5/6 mutant, we have also done experiments to determine TE reactivation in roots of a subset of TEs known to have a reduction in H3K27me1 level in the atxr5/6 mutant. In roots, TE reactivation is small or absent. For some TEs in orc1a-2, atxr5/6 and the triple mutant some TE expression is detected but these differences are not as high as for the robust atxr5/6 phenotype (Fig. 5c).**

-In general, can the two alleles that are used (orc1a-2 and orc1b-2) be compared vis-a-vis penetrance? What are the mutations that generated these alleles? Can it be said that are they equally affecting protein function? If this cannot be tested rigorously, it remains challenging to make strong conclusions regarding specific functions in which one of the genes is 'more important' than the other. In relation to this: can a double mutant be made (i.e. orc1a-2, orc1b-2)? If so, what is the phenotype?
**R: We have not formally studied penetrance. The mutant alleles are produced by T-DNA insertion in the middle of the genes (see methods and Supplementary Fig S3). In none of them we can detect the presence of full-length ORC1 mRNAs. Also, based on the location of the T-DNA insertions in both genes, a putative truncated peptide produced (consisting of the N-terminal amino acid sequences upstream of the T-DNA inserted) will not contain the full AAA+ domain. Therefore, we can conclude that a functional full-length protein is not produced. We have tried to generate a double mutant by crossing, but after testing many of the progeny seedlings, we have not been able to identify a double mutant. This may be due lethality, at some stage of development, or due to the linkage of the two genes in Chrom 4.**

-To what extent does the fact that many other DNA replication genes show duplications (the authors mention Cdc6 and Cdt1) influence potential interpretations towards functionalization? In the example that is used (budding yeast), this does not appear to be the case, and Orc1 stands out as having a diverged paralog (Sir3). How could this be interpreted?
**R: You are right that in yeast it is only Orc1 and Sir3. We do not have yet compelling functional information about the duplicated CDC6 and CDT1 genes. We know that a knock-down of CDC6 genes are viable but a knock-out has not been identified, suggesting that it**

**may be lethal. Since ORC1 is at the initial stages of pre-RC assembly, it is reasonable to consider that a defect at that early stage should not be influenced by the presence of other proteins (CDC6 and CDT1) generated from duplicated genes, but this is a simple speculation. We hope that the reviewer would agree that expanding the work in this direction may be the subject of study in the future.**

-Page 3, first paragraph of results: '..orc1b expression co-localizes…'. Strange wording, please rephrase.
**R: Changed to "… ORC1b expression is detected in proliferating cells …**

-Page 5, line 3: '...confirming that Orc1b was loaded in G2..'. Loaded seems to be potentially confusing terms here: thes experiments only reveal localization patterns, whereas loading, in the context of ORC, might be interpreted to mean something more specific. Please rephrase.
**R: We agree. This has been changed to: "… confirming that ORC1b accumulates in mid/late G2…"**

-Page 5, third paragraph: ' live imaging…'. These findings do to reinforce that Orc1 is degraded per se. Please rephrase.
**R: Changed to: "… Live imaging experiments confirmed this sincie all the ORC1b-GFP… "**

-Page 5, fourth paragraph: these experiments suggest that proteosomal degradation might influence Orc1 protein levels, but do not 'establish' a direct link. The effect can conceivably be rather indirect. Rephrase statements.
**R: Changed to: "… These results, together with the live-imaging and EdU-labeling results, suggest that proteasomal degradation influences ORC1b, but not ORC1a, protein levels and that ORC1b is targeted …".**

-Page 6, first paragraph: what is the conclusion of these experiments?
**R: We have added: "These results suggest that loss of either ORC1a or ORC1b do not lead to obvious phenotypic changes in root development under normal growth conditions."**

-Page 6, second paragraph: the section describing the BrdU/EdU pulses is hard to digest in its current form, please rewrite. In terms of experiments: what would the effect of a classical, strong S-phase mutant be in such an analysis, for reference? Can such a mutant be tested?
**R: We are not aware of the existence of such strong mutants, which are very likely to be embryo lethal. In case of being viable, it might have a delayed S-phase progression, e.g., need of a longer chase time between the pulses to reach ~0% of nuclei having the two analogues. However, we think that an exhaustive search for such mutant could be, at the present time, a difficult task since they should be first identified in the collection and then tested. We hope the reviewer agrees that this approach could be left for future studies. We have rewritten this section, as requested, trying to clarify the description.**

-Page 7, first paragraph: it would be very interesting if the authors can add more insight into this part. Is heterochromatin impaired during all cell cycle phases? Is it different in G1 vs S/G2? What is the rationale about linking orc1a to atxr5/6? Is there a direct functional connection? Related: in Figure 5: in Figure 5B, please include in the same analysis the single mutants (and the orc1-a without Orc1a-GFP).

**R: Based on the expression domain of ORC1a, largely confined outside the root meristem where cell proliferation occurs, it seems that ORC1a plays its role preferentially in the transition/elongation zones, where cells are endocycling. As you know, these cells only have a gap phase in between the S-phase of the endocycle. We have changed the first sentence of this paragraph to make more explicit the rationale behind trying to find a link between ocr1a-2 and atxr5/6 mutants. We have genetic data (the triple orc1a-1,atxr5/6 mutant) suggesting they act in the same pathway, since its phenotype is indistinguishable from that of the orc1a-2 and atxr5,atxr6 mutants.**
**The new Fig. 5a has been modified and all experiments included in a single panel (two independent experiments/measurements of the wild type Col-0 have been included).**

**Reviewer #3 (Remarks to the Author):**

This study presents a comprehensive overview of the expression/localization dynamics of the ORC1A and ORC1B proteins in plant tissues, cells and subnuclear compartments, from which it is derived that they have different functions. ORC1B plays a role in DNA replication in defining the origin of replications, while the role of ORC1A has diverged and appears to be important for the maintenance of the repressive histone mark H3K27me1. Therefore, this work provides the first detailed characterization in plants of the different functions of these proteins, and as such, it represents an important contribution to our understanding of DNA replication in eukaryotes. The conclusions presented are justified, and the quality of the work is very good.

Please see below for comments that may improve the manuscript.

1. Characterization of the single orc1A and orc1b mutants clearly validates that these proteins have some independent functions in plants. However, it would have been interesting to create a double mutant to see if these proteins play partially overlapping roles in some instances. Creating such a double mutant in Arabidopsis and re-doing some of the analyses would take many months, so I don't think doing this work is a requirement for publication. However, if the double mutant is available, some basic characterizations of its phenotype(s) could be presented to answer the question of partial overlap.
**R: We have tried to generate a double mutant by crossing, but after testing many of the progeny seedlings, we have not been able to identify a double mutant. This may be due lethality, at some stage of development, or due to the linkage of the two genes in Chrom 4. In any case, ORC1b is primarily expressed in proliferating cells (although also in endocycling cells) whereas ORC1a is expressed exclusively in endocycling cells and localized to the chromocenters. The single mutants have distinct phenotypes. Therefore, even in the cells where the two proteins are expressed (endocycling cells), it seems that the ORC1b protein cannot complement the heterochromatin phenotype of the orc1a-2 mutant.**

2. The effect of the orc1a mutant on lowering H3K27me1 levels is interesting, and could benefit from being characterized a little bit further. For example, atxr5 atxr6 double mutants show re-replication phenotypes on FACS profiles. Do we see the same phenotypes for the orc1a mutant? What about transcriptional derepression in heterochromatin for the orc1a mutant? If similar to the atxr5 atxr6 mutant, then we could expect some transposable elements to be reactivated in the orc1a mutants?
**R: These are certainly interesting questions. We have done new flow cytometry experiments to address this point. Neither the orc1a-2 nor the atxr5,atxr6 mutant show a clear re-replication phenotype in nuclei isolated from roots (new Fig 5b). The re-replication phenotype of the atxr5,atxr6 double mutant is detected in 4-week leaves (Jacob et al., 2010). Thus, we also looked at the flow cytometry profiles in 4 week-old leaves and confirmed that the atxr5,atxr6 mutant shows the typical phenotype but that the orc1a-2 seems to be wild type. This is consistent with the absence of any detectable signal of ORC1a-GFP in leaves (young or old leaves). Therefore, it seems that the loss of ORC1 or ATXR5/6 proteins does not lead to a re-replication phenotype in roots, consistent with a recent report (Potok et al., 2022,**

**doi: 10.1073/pnas.2115570119 ). It is important to keep in mind that the ploidy level reached in roots is not as high as in old leaves. We have included this analysis in a new Fig. 5b and a new Suppl. Fig. 7, its description in the Results and a short comment in the Discussion.**
**Regarding the TE derepression observed in the atxr5/6 mutant, we have also done experiments to determine TE reactivation in roots of a subset of TEs known to have a reduction in H3K27me1 level in the atxr6/6 mutant. In roots, TE reactivation is negligible or absent in the orc1a-2 mutant, as well as in the atxr5/6 mutant (in this case except for the Tsi case). This is now included as a new Fig 5c.**

Minor edits:

1. Fig. 1H and 1I: The name used for the upper part of the root in the Figure is EZ+DZ. However, the name for this zone used in the text is TZ+EZ.
**R: Corrected**

2. I would slightly alter the following sentence to clarify the meaning, assuming the edits do not change the meaning of the sentence:
"The nuclear pattern of ORC1b-GFP was completely different, with a preferential localization in the proliferation zone (50.1%) and in the transition and elongation zone (51.5%; Fig. 1i)."

to this sentence:

"The root-specific pattern of ORC1b-GFP localization was completely different compared with ORC1A-GFP, as shown by a majority cells containing ORC1B in both the proliferation zone (50.1%) and in the transition and elongation zone (51.5%; Fig. 1i)"
**R: Thank you for the suggestion. The sentence has been changed.**

3. "The patchy pattern of ORC1b protein in proliferating cells supports a cell cycle regulation."

I would change to:

"The patchy pattern of ORC1b in proliferating cells suggests that expression of the protein is regulated during the cell cycle."
**R: This sentence has been changed.**

4. Fig. 1 is missing labels for panels E, F, and G
**R: This has been corrected.**

5. Legend of Fig. 1. An arrowhead for the mature guard cells is mentioned in the legend, but omitted on the figure. It's also hard to distinguish the solid color line indicating the mean and SEM (?) on panels H and I. Maybe changing the color of the line to black would help. Finally, it says at the bottom of the legend that the most common patterns in panels H and I are shown in bold. Since green and blue colors are used, I would refer to the colors instead.
**R: We actually meant "arrows", now corrected. The solid lines have been changed to black, to visualized them better. The legend has been also corrected.**

6. Fig. 2. In panel C, I am not sure what the star indicates (localization marker?), and there is no mention of it in the legend. There is also a typo in the middle of the legend, with “Th” missing an “e”.
**R: In the legend to panel c, we have added: “as illustrated by the cell labeled with an asterisk.”. The typo has been corrected.**

7. Supplementary Fig. 3. Typically, for these T-DNA validation experiments, primer pairs will also be used to assess expression upstream and/or downstream of the T-DNA expression, to see if a partial transcript is still expressed. This is particularly important if the T-DNA is located in the promoter or near the 3’end of a gene, which is not the case here. However, it is still informative to see the impact of T-DNA on the expression of any part of a gene.
**R: This analysis has been now included in the new Suppl. Fig. 2**

Also, it’s not clear on panel A what part of the gene is shown. Does it go from the start codon to the stop codon, with the 3’UTR indicated by a white box at the end of the genes? Also, it would be good to remind the readers in the legend that the ORC1A and ORC1B genes do not have introns, thus confirming that the T-DNA insertions are in exons.
**R: See comment above, new Fig. 2**

8. Supplementary Fig. S6. This figure is missing SEM and statistics.
**R: A new Suppl. Fig. 5 has been included with appropriate stats (3 biological replicates, each with 2 technical replicates). The results are consistent with the preliminary data.**

9. Supplementary Fig. S7. It would be informative to include the correlation coefficients on the panels.
**R: We have included the regression lines in the new Suppl. Fig. 6 and in the new Suppl. Table 1, b ut not the correlation coeficients, which are not provided using the Deming (Model II) regression.**

Reviewer #1 (Remarks to the Author):

I acknowledge that the manuscript has been substantially revised with new data , a revised text according to the suggested changes and figures. These are important revisions improving the quality of the manuscript and robustness of some, but not all, conclusions.
I still have a major concern concerning one of the central statement (point 1) and requests for clarifying the presentation of quantitative experiments (points 2, 3) and some text corrections (point 4). I believe that addressing these issues are important to provide exemplary data at the level expected for this journal.

1) Role of ORC1a on heterochromatin
The differentiated role of ORC1a on heterochromatin is deduced from the following statements
1a) "ORC1a localizes to chromocenters"
1b) Despite this localization, the ORC1a mutation does not impact the amount of heterochromatin
1c) ORC1 influences H3K27me1 levels at chromocenters
However, not all of them are clearly showed which is problematic as this is a key message from the paper.
1a) "ORC1a localizes to chromocenters": this is a statement based on the punctuate localization of ORC1a-GFP (Fig 1, Fig 2). But formally speaking, that the punctuate structures are chromocenters is not shown. Images showing co-localisation with chromocenters using a DNA (or H3) counterstaining is necessary.

1b) Despite this localization, the ORC1a mutation does not impact the amount of heterochromatin: this is a statement from the authors in their rebuttal. But no data are shown (neither images, nor quantifications). This is an important result enabling to discuss the interesting fact that ORC1a would contribute regulating H3K27me1 levels (next point) but downstream of chromocenter formation (?). The authors have apparently produced this data and it would be relevant to present them.

1c) ORC1a influences H3K27me1 levels at chromocenters (Fig5, Fig S7). This conclusion is reached following immunostaining experiments against H3K27me1 and the quantification of immunosignals across chromocenters (Fig 5 and S7),. In my former evaluation, I raised the issue that such quantification should be normalized, in the same way than quantitative RT-PCR or quantitative western blot are done, that is, using an internal control as reference. The authors reply that this is not necessary since the H3K27me1 measurements have been done on the exact same nuclei where measurements for H3K9me2 were done. I see three major problems with that:
(i) the method indicates that both histone modifications have been immunostained using rabbit primary antibodies and detected with anti-rabbit secondary antibodies both conjugated with "488" (presumably Alexa488). Thus, either the experiments have been done separately and this would be the equivalent of doing an RT-PCR with gene A on one RNA sample and an RT-PCR on the reference gene (eg Actin) on a different RNA sample. Or, the methods are wrong and both histone modifications have been co-immunostained using two different primary and secondary antibodies with distinct fluorophoores. In the first case, the comparisons fig 5, and the scatter plots Fig S7 are not valid. In the later case, the methods should be corrected, and my second point should be considered.
(ii) using H3K9me2 as internal control is a post-hoc decision and creates a circular argument. There was/is not reason to consider that this mark does not change in the orc1a mutant. Hence no reason to use it as reference to compare with H3K27me1 levels. It creates a circular argument in that, to conclude that H3K9me2 levels do not change in the orc1a mutant, a proper quantification with an internal reference is required.
(iii) according to the methods and the annotation of the data in supplementary table 1, it seems that the approach used is the quantification of immunostaining signal levels along a line drawn over single chromocenters . This approach is certainly not canonical, nor is it not justified (not to say, not valid) for the following reasons: measurements done only in a tiny (10%?) fraction of the chromocenter, variability is expected due to the variable positioning of the line with regard to the chromocenter (middle, side) which will not have a homogenous signal intensity; biaised measurements are expected with variable size of chromocenters.
One possible example of bias: if orc1a has smaller chromocenters than wild-type but with the same amount of H3K27me1, the line-based measurement will artificially give lower H3K27me1

signals in the mutant.

In conclusion: (1) the measurements should be done on the entire chromocenter nuclei (or nuclei, probably easier) , ie over areas, (2) the measurements should be normalized using DAPI or anti-H3 signals (different colors), (3) representative images of immunostaining should be shown, (4) the result should be confirmed with an independent method, ie using quantitative western blots

2) Quantification of ORC1b degradation
The text revisions and the new experiments using different proteasome inhibitors in microscopy imaging and western blot are strengthening the conclusions. Revisions efforts are much appreciated. Yet, and because studies published in such a high-quality journal should be exemplary in terms of replication of observations and measurements, please:
- Provide the number of roots scored showing high vs low GFP signals (in the epidermis) to justify the "representative" images (representative out of how many? Is there any variation?)
- Indicate the number of western blot replicates used for graph 3c
- Indicate the type of statistical test performed on the measurements.
- Raw data for the graph 3c should be provided in a supplementary table.
- How were the western blot signals quantified and normalized for the graph figure 3c?

3) Quantification of ORC1b-EdU co-labeling
Figure 4 b and legend "For each chase time point the percentage of colabeled nuclei in several biological replicates of each genotype was plotted. At least, 100 nuclei were scored for each time point."
- Please indicate the number of replicates ("several") , likely 2-3 /time point according to the graph
- Please provide the scoring data for each replicate in a supplementary table
- Explain in the legend that each colored point correspond to one replicate, and that the line correspond to ..what ? (mean?)

4) Text corrections
L246-247: Fig5a left panel is indicated for both orc1a and orc1b
Figure 5 legend: (a) is missing
Supplementary Table 1 (H3K27me1, H3K9me2 intensity, number of chromocenters): spelling mistake "intensity" (n is missing)
L479-481 (Methods) : Measurements of fluorescent intensity.
- Indicate which software was used
- Does this mean that fluorescence intensity in chromocenters was measured using one line intersecting a chromocenter? This method is unconventional as usually the whole heterochromatin/chromocenter region should be measured as a circular/spheroid ROI.
L765-766 (Fig 4 legends) , orc mutants should be in italics
L436 what is described is immunostaining, not immunohistochemical assays (which would be for instance a GUS assay)
L441 is it really 20mg/mL driselase? This sounds like really a lot (10x more than usual)
L 445-446: H3K9me2, H3K27me2, GFP and BrdU are not primary antibodies. Please correct for : anti-H3K9me2, anti-H3K27me3, anti-GFP, anti-BrdU
L448-449: the secondary antibody description is incomplete. The fluorophore is missing. 488 alone does not exist, it has to be Alexa488 (or other fluorophore?). But really correct is: Donkey anti-rabbit IgG- Alexa488 (and similar for the others)

5) Revisions acknowledged for
- Scoring of ORC1a-ORC1b distribution in root zones (Fig 1i, 1j): My comment referred to the scoring showed below the graph according to the proliferation zone, the elongation zone and the differentiation zone. Not relative to euchromatin and heterochromatin where, as the authors replied in their rebuttal, a GUS enzymatic assay is not appropriate. My comment in the first evaluation was coming from the numbers below the graphs, for instance: 1.6% Euchromatin pattern in a TZ+EZ region was difficult to interpret without knowing the number of nuclei and only knowing that 5 roots were scored. It suggested in the end only a couple of nuclei were found with this pattern, in this region among 5 roots, which does not allow to appreciate the level of replication.

It was thus justified to increase the replication level ( higher number of roots and nuclei) as 1000 nuclei allow to give sense to small percentages of distribution per root zone. The issue is thus clarified
- Complementation of orc1b Aphidicolin sensitivity ( root length phenotype) by ORC1b-GFP: My apologies for the typo (indeed ORC1b-GFP and not ORC2-GFP) and thank you for the new graph and analysis figure 4d commented in the text. It indeed shows a partial complementation.

Reviewer #2 (Remarks to the Author):

This revised manuscript by Vergara et al describes an initial functional query into the roles of the two ORC1a and ORC1b genes in Arabidopsis thaliana.

The authors have taken care to address the points I have earlier reached, and in principle have done a good job. However, this reviewer remains uncertain as to the mechanistic insight that can be currently gleaned from this study. Despite a commendable effort, the experiments that have been performed/added in this revised version do not significantly alter my opinion in that matter. Especially the role of ORC1a remains rather unclear, apart from an observed effect on one heterochromatin marker- but no further functional consequence known as of yet. Further, the experiments that are now added make it less obvious as to the function consequences of the removal of this factor. More comprehensive analysis of heterochromatin function remains needed.

If this factor really acts 'uniquely' in heterochromatin formation this is in principle expected to be reflected in protein structure/domain organisation.This reviewer asked for a detailed analysis of any difference between 1a and 1b, but the current info provided is rather sparse, and makes one believe these two factors are (near) identical? If not, the conclusion one is left with that the different effects of 1A/1B are primarily set by expression timing. Is that the idea of the authors? If so, do 1B mutants also have heterochromatin defects in affected cells? What would happen if 1b is expressed under 1a regulation? Also, knowing whether a double mutant is lethal remains important to reveal. Is the idea that orc1b is not essential due to cdc6 taking over, or because of orc1a?

In its current form, this reviewer remains ambiguous about the mechanistic insight gained in this study.

Minor points:

Especially the abstract and introduction sections currently suffer from awkward/incorrect writing. Please make sure to improve. Some examples (note: not an exhaustive list)

- line 39. "however, little is known about...of multicellular organisms". This is a misplaced and unnecessary sentence.
- line 41. "Arabidopsis encodes..." change to : "The Arabidopsis genome encodes for.."
- line 42-43: " ...although showing ...., they..." Who is they here? This is a grammatically problematic sentence
- line 77: '" ..in relation to cell cycle lags..", should be changed, to something like " in relation to cell cycle progression lags..."
-line 99. 'Their promoters are different.., and encode for" promoters don't encode for genes, so please correct the sentence.
Please carefully proofread the manuscript.

Reviewer #3 (Remarks to the Author):

All my comments have been addressed by the authors in the revised version of the manuscript.

**Reviewer #1 (Remarks to the Author):**

I acknowledge that the manuscript has been substantially revised with new data , a revised text according to the suggested changes and figures. These are important revisions improving the quality of the manuscript and robustness of some, but not all, conclusions.
I still have a major concern concerning one of the central statement (point 1) and requests for clarifying the presentation of quantitative experiments (points 2, 3) and some text corrections (point 4). I believe that addressing these issues are important to provide exemplary data at the level expected for this journal.

1) Role of ORC1a on heterochromatin
The differentiated role of ORC1a on heterochromatin is deduced from the following statements
1a) "ORC1a localizes to chromocenters"
1b) Despite this localization, the ORC1a mutation does not impact the amount of heterochromatin
1c) ORC1 influences H3K27me1 levels at chromocenters
However, not all of them are clearly showed which is problematic as this is a key message from the paper.

1a) "ORC1a localizes to chromocenters": this is a statement based on the punctuate localization of ORC1a-GFP (Fig 1, Fig 2). But formally speaking, that the punctuate structures are chromocenters is not shown. Images showing co-localisation with chromocenters using a DNA (or H3) counterstaining is necessary.
**R: Yes, the statement was based on the punctate appearance of ORC1-GFP proteins that was similar to the appearance of DAPI-stained nuclei. To formally show if they colocalize with chromocenters we carried out experiments to immunodetect ORC1-GFP (using either ORC1a-GFP and ORC1b-GFP-expressing roots) together with H3K9me2, the well-known and accepted typical marker of heterochromatic chromocenters. The results are included the new panel Fig 1k, showing images of nuclei with the three different patterns shown in Fig. 1i and Fig 1j . We found that ORC1a-GFP protein colocalizes with the typical heterochromatic mark H3K9me2, demonstrating that the punctate pattern of ORC1a-GFP corresponds to the chromocenter pattern.**

1b) Despite this localization, the ORC1a mutation does not impact the amount of heterochromatin: this is a statement from the authors in their rebuttal. But no data are shown (neither images, nor quantifications). This is an important result enabling to discuss the interesting fact that ORC1a would contribute regulating H3K27me1 levels (next point) but downstream of chromocenter formation (?). The authors have apparently produced this data and it would be relevant to present them.
**R: This is in fact a speculation since we have not formally addressed the temporal relationship of ORC1a localization with chromocenter formation, which would require a whole series of new experiments. In any case, to address the reviewer's request we have quantified the relative amount of heterochromatin by determining the relative heterochromatin fraction (RHF), as used in Soppe et al. (2002) and Tessadori et al. (2004), now included in the main text and in the new Fig. 5a.**

1c) ORC1a influences H3K27me1 levels at chromocenters (Fig5, Fig S7). This conclusion is reached following immunostaining experiments against H3K27me1 and the quantification of

immunosignals across chromocenters (Fig 5 and S7),. In my former evaluation, I raised the issue that such quantification should be normalized, in the same way than quantitative RT-PCR or quantitative western blot are done, that is, using an internal control as reference. The authors reply that this is not necessary since the H3K27me1 measurements have been done on the exact same nuclei where measurements for H3K9me2 were done. I see three major problems with that:

(i) the method indicates that both histone modifications have been immunostained using rabbit primary antibodies and detected with anti-rabbit secondary antibodies both conjugated with “488” (presumably Alexa488). Thus, either the experiments have been done separately and this would be the equivalent of doing an RT-PCR with gene A on one RNA sample and an RT-PCR on the reference gene (eg Actin) on a different RNA sample. Or, the methods are wrong and both histone modifications have been co-immunostained using two different primary and secondary antibodies with distinct fluorophoores. In the first case, the comparisons fig 5, and the scatter plots Fig S7 are not valid. In the later case, the methods should be corrected, and my second point should be considered.

(ii) using H3K9me2 as internal control is a post-hoc decision and creates a circular argument. There was/is not reason to consider that this mark does not change in the orc1a mutant. Hence no reason to use it as reference to compare with H3K27me1 levels. It creates a circular argument in that, to conclude that H3K9me2 levels do not change in the orc1a mutant, a proper quantification with an internal reference is required.

(iii) according to the methods and the annotation of the data in supplementary table 1, it seems that the approach used is the quantification of immunostaining signal levels along a line drawn over single chromocenters . This approach is certainly not canonical, nor is it not justified (not to say, not valid) for the following reasons: measurements done only in a tiny (10%?) fraction of the chromocenter, variability is expected due to the variable positioning of the line with regard to the chromocenter (middle, side) which will not have a homogenous signal intensity; biaised measurements are expected with variable size of chromocenters. One possible example of bias: if orc1a has smaller chromocenters than wild-type but with the same amount of H3K27me1, the line-based measurement will artificially give lower H3K27me1 signals in the mutant.

**R: This comment appears to be a misinterpretation derived from our unfortunate error based on (1) a figure in the first submission showing the signal across chromocenters in a few nuclei, which was already eliminated in the revised version, and (2) from not deleting the corresponding sentence in the revised text (methods). We must emphasize that we measured the signals in individual chromocenters considering the entire area covering each of them (as correctly stated now in the Methods) and not using any line across them. Even in the original submission, the line was used for the figure (qualitative) and not for the measurements of individual chromocenters.**

**R i) Yes, the methods were wrong. Both histone modifications have been co-immunostained using two different primary and secondary antibodies with distinct fluorophores. We used the primary antibody anti-H3K9me2 (Abcam ab1220) in combination with the secondary antibody Donkey anti-Mouse IgG-Alexa Fluor 555 (A-31570), and the primary antibody anti-H3K27me1 (Millipore 07-448) with the secondary antibody Donkey anti-Rabbit IgG-Alexa Fluor 488 (A-21206). The text has been corrected in the methods section.**

**R ii) We consider that our approach is not post hoc, as indicated. Once we observed the localization of ORC1a-GFP to chromocenters, we carried out in the mutants (and Col-0) an analysis of the H3K9me2 mark, widely accepted as a typical (and quantitative) heterochromatic mark. We found that this signal in the mutants was indistinguishable**

**from that of the wild type. Thus, we could complete the experiment described in the manuscript by immunostaining both H3K9me2 and H3K27me1 in the same nuclei to measure both signals in each individual chromocenter. This is absolutely similar to what is done (or sometimes assumed) in the RT-qPCR experiments mentioned by the reviewer: one takes one gene as a putative reference, then measures the mRNA under different conditions, and in case that it does not change it can be used later as reference gene. Therefore, we argue that our approach is equally valid to that proposed by the reviewer. In fact, a H3 signal, as proposed, may vary between eu- and heterochromatin, in particular due to differences in the relative amount of H3.1 and H3.3 (Stroud et al., 2012; Wollman et al., 2012). For obvious reasons, this is too wordy to include in a manuscript and we are convinced that it is a valid way of proceeding. There is no assumption, as indicated by the reviewer. We hope that our argument is finally accepted as a valid method for quantitative analysis of the H3K27me1 content in chromocenters.**
**R iii) As indicated earlier, we have done it by measuring the integrated signal of an area covering the entire chromocenter. The text has been corrected in the methods section stating that measurements have been done using an entire ROI (not along a line).**
**We hope that it is clear now that we have measured chromocenter intensity signals properly.**

In conclusion: (1) the measurements should be done on the entire chromocenter nuclei (or nuclei, probably easier) , ie over areas,
**R: This is how measurements have been done, over areas, not along a line (see above)**
(2) the measurements should be normalized using DAPI or anti-H3 signals (different colors),
**R: We consider that our approach is as valid as the one suggested by the reviewer (see above)**
(3) representative images of immunostaining should be shown,
**R: We have included representative images of the immunostaining of ORC1-GFP plus H3K9me2 (new Fig 1k), and of the immunostaining of both H3K9me2 and H3K27me1 in the same chromocenter (New Fig. 5c).**
(4) the result should be confirmed with an independent method, ie using quantitative western blots
**R: We believe that there are objective reasons to consider that the proposed experiment may not be appropriate. It has been reported that the atxr5,atxr6 double mutant shows a decrease of only ~20% in the total amount of H3K27me1 (Jacob et al., 2009, NSMB 6, 763-768, Suppl Fig 3, see below).**

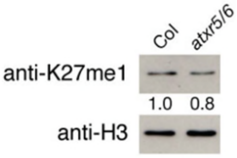


**Supplementary Fig. 3.** Western blot analysis of H3K27me1. Quantization was performed using ImageJ.

**It must be emphasized that the Western blot was carried on extracts of entire seedlings (or leaves) where ATXR5/6 is expressed. This is by far not comparable to the situation of ORC1a that is expressed in a small number of cells in the transition zone of the root while H3K27me1 is present in the vast majority of cells in the root. Therefore, changes in the total amount of H3K27me1, due to the orc1a-2 mutation, are very unlikely to be feasibly detected by a Western blot analysis, as requested.**

**We hope that the reviewer accepts our argument and the reasons to analyze in detail by immunostaining the effects of the orc1a-2 mutation, but not by Western blot.**

2) Quantification of ORC1b degradation
The text revisions and the new experiments using different proteasome inhibitors in microscopy imaging and western blot are strengthening the conclusions. Revisions efforts are much appreciated. Yet, and because studies published in such a high-quality journal should be exemplary in terms of replication of observations and measurements, please:
- Provide the number of roots scored showing high vs low GFP signals (in the epidermis) to justify the “representative” images (representative out of how many? Is there any variation?)
**R: The number of roots used in each case of panels 3a and 3b are now indicated in the legend.**

- Indicate the number of western blot replicates used for graph 3c
**R: Western blot analysis shown in Fig 3c consisted of two independent biological replicates (with technical replicates).**

- Indicate the type of statistical test performed on the measurements.
**R: t-Student**

- Raw data for the graph 3c should be provided in a supplementary table.
**R: For your information, we provided these data in a the new Supplementary Table 1 that contains the raw data used to generate each figure.**

- How were the western blot signals quantified and normalized for the graph figure 3c?
**R: Measurements were done using Fiji, as indicated in the Summary Report. We have now indicated in the figure the band quantified as ORC1 and the band used to normalize the measurements. Note that, as indicated in the legend, the protein pattern is different for ORC1a and ORC1b analysis because extracts were prepared from roots and seedlings, respectively.**

3) Quantification of ORC1b-EdU co-labeling
Figure 4 b and legend “For each chase time point the percentage of colabeled nuclei in several biological replicates of each genotype was plotted. At least, 100 nuclei were scored for each time point.”
- Please indicate the number of replicates (“several”) , likely 2-3 /time point according to the graph
**R: Please, see the legend and Supplementary Table 1.**

- Please provide the scoring data for each replicate in a supplementary table
**R: The data requested are provided for your information in the new Supplementary Table 1 that contains the raw data used to generate each figure.**

- Explain in the legend that each colored point correspond to one replicate, and that the line correspond to ..what ? (mean?)
**R: It seems that the data displayed in this panel were not be sufficiently clear. Therefore we have decided to display the data as a bar graph, including the stats and the data points,**

**which seems to be clearer.**

4) Text corrections
L246-247: Fig5a left panel is indicated for both orc1a and orc1b.
**R: Yes, it was right. Data reporting H3K27me1 distributions for both orc1a-2 and orc1b-2 (together with those of other genotypes) are displayed in the left group of Fig. 5a, as indicated in the text. Also data for H3K9me2 are displayed in Fig 5a (the group on the right, as indicated).**

Figure 5 legend: (a) is missing.
**R: Sorry, but we are not sure what the reviewer meant by indicating that (a) is missing. "a," appeared in the legend.**

Supplementary Table 1 (H3K27me1, H3K9me2 intensity, number of chromocenters): spelling mistake "intensity" (n is missing)
**R: Corrected.**

L479-481 (Methods) : Measurements of fluorescent intensity.
- Indicate which software was used
**R: We used Fiji. The software for data acquisition and image analysis was specified in the Reporting Summary requested by the journal and provided with this submission.**

- Does this mean that fluorescence intensity in chromocenters was measured using one line intersecting a chromocenter? This method is unconventional as usually the whole heterochromatin/chromocenter region should be measured as a circular/spheroid ROI.
**R: We repeat here what we mentioned earlier. This comment appears to be a misinterpretation derived from an unfortunate error based on (1) a figure in the first submission showing the signal across chromocenters in a few nuclei, which was eliminated in the revised version, and (2) from not deleting the corresponding sentence in the revised text. We must emphasize that we measured the signals in individual chromocenters considering the entire area covering each of them (as correctly stated now in the revised Methods) and not using any line across them. The line was used for the figure in the original submission and not for the measurements of individual chromocenters. In any case, that figure was deleted from the revised version and consequently we have corrected the sentence in the methods section to clarify this point.**
**The text has been corrected in the methods section stating that measurements have been done using an entire ROI (not along a line).**

L765-766 (Fig 4 legends) , orc mutants should be in italics
**R: Thank you. Corrected.**

L436 what is described is immunostaining, not immunohistochemical assays (which would be for instance a GUS assay)
**R: Thank you. Corrected.**

L441 is it really 20mg/mL driselase? This sounds like really a lot (10x more than usual)
**R: We are using driselase at 2% (that is 20mg/ml), as described in Sauer and Friml (2010).**

L 445-446: H3K9me2, H3K27me2, GFP and BrdU are not primary antibodies. Please correct for : anti-H3K9me2, anti-H3K27me3, anti-GFP, anti-BrdU
**R: Thank you. Corrected.**

L448-449: the secondary antibody description is incomplete. The fluorophore is missing. 488 alone does not exist, it has to be Alexa488 (or other fluorophore?). But really correct is: Donkey anti-rabbit IgG- Alexa488 (and similar for the others)
**R: You are right. We have corrected the text.**

5) Revisions acknowledged for
- Scoring of ORC1a-ORC1b distribution in root zones (Fig 1i, 1j): My comment referred to the scoring showed below the graph according to the proliferation zone, the elongation zone and the differentiation zone. Not relative to euchromatin and heterochromatin where, as the authors replied in their rebuttal, a GUS enzymatic assay is not appropriate. My comment in the first evaluation was coming from the numbers below the graphs, for instance: 1.6% Euchromatin pattern in a TZ+EZ region was difficult to interpret without knowing the number of nuclei and only knowing that 5 roots were scored. It suggested in the end only a couple of nuclei were found with this pattern, in this region among 5 roots, which does not allow to appreciate the level of replication.
It was thus justified to increase the replication level ( higher number of roots and nuclei) as 1000 nuclei allow to give sense to small percentages of distribution per root zone. The issue is thus clarified
- Complementation of orc1b Aphidicolin sensitivity ( root length phenotype) by ORC1b-GFP: My apologies for the typo (indeed ORC1b-GFP and not ORC2-GFP) and thank you for the new graph and analysis figure 4d commented in the text. It indeed shows a partial complementation.
**R: Thank you.**

**Reviewer #2 (Remarks to the Author):**

This revised manuscript by Vergara et al describes an initial functional query into the roles of the two ORC1a and ORC1b genes in Arabidopsis thaliana.

The authors have taken care to address the points I have earlier reached, and in principle have done a good job. However, this reviewer remains uncertain as to the mechanistic insight that can be currently gleaned from this study. Despite a commendable effort, the experiments that have been performed/added in this revised version do not significantly alter my opinion in that matter. Especially the role of ORC1a remains rather unclear, apart from an observed effect on one heterochromatin marker- but no further functional consequence known as of yet. Further, the experiments that are now added make it less obvious as to the function consequences of the removal of this factor. More comprehensive analysis of heterochromatin function remains needed.
If this factor really acts 'uniquely' in heterochromatin formation this is in principle expected to be reflected in protein structure/domain organisation.This reviewer asked for a detailed analysis of any difference between 1a and 1b, but the current info provided is rather sparse, and makes one believe these two factors are (near) identical? If not, the conclusion one is left with that the different effects of 1A/1B are primarily set by expression timing. Is that the idea of the authors? If so, do 1B mutants also have heterochromatin defects in affected cells? What would happen if 1b is expressed under 1a regulation? Also, knowing whether a double mutant is lethal remains important to reveal. Is the idea that orc1b is not essential due to cdc6 taking over, or because of orc1a?

In its current form, this reviewer remains ambiguous about the mechanistic insight gained in this study.

**R: Thank you for recognizing our efforts in addressing your points. We definitely agree that in the present study we have uncovered a distinct role of the ORC1 proteins although several questions still remain unanswered and would require extensive work and a whole new series of experiments. We have pointed out to the major differences between the two at the beginning of our description. ORC1a and ORC1b are identical (87% in amino acid sequence), except in the 50-60 N-terminal residues where it is conceivable that one or more motifs exist that confer specific functions. These functions do not seem to be related to differences in expression timing, in fact in expression domains within the root, at least in cells located in the proximal part of the meristem and in the transition zones. Since these cells express both proteins and we can unambiguously identify different phenotypes in the orca1-2 and orc1b-2 single mutants, we can conclude that the defects observed in S-phase progression and heterochromatic H3K27me1 marks, respectively, are not complemented by the other ORC1 form that is wild type. As we show in Fig. 5b, the orc1b-2 mutant is normal in the amount of H3K27me1. One experiments that remains to be done is to express ORC1a under the ORC1b promoter and focus in the meristematic region and in the S-phase progression. Also, it would be important to explore other swapping experiments, including small deletions and/or point mutations within the N-terminal domains of both ORC1a and ORC1b. Generating all these new lines and doing the necessary experiments, which could constitute by themselves a whole new project, will take months and we think that they are beyond the scope of this initial manuscript. We do hope that the reviewer can accept this argument.**

Minor points:

Especially the abstract and introduction sections currently suffer from awkward/incorrect writing. Please make sure to improve. Some examples (note: not an exhaustive list)
- line 39. "however, little is known about...of multicellular organisms". This is a misplaced and unnecessary sentence.
**R: Corrected**

- line 41. "Arabidopsis encodes..." change to : "The Arabidopsis genome encodes for.."
**R: Corrected**

- line 42-43: " ...although showing ...., they..." Who is they here? This is a grammatically problematic sentence
**R: Corrected**

- line 77: "' ..in relation to cell cycle lags..", should be changed, to something like " in relation to cell cycle progression lags..."
**R: Corrected.**

-line 99. 'Their promoters are different.., and encode for" promoters don't encode for genes, so please correct the sentence.
**R: Corrected**

Please carefully proofread the manuscript.
**R: Done**

**Reviewer #3 (Remarks to the Author):**

All my comments have been addressed by the authors in the revised version of the manuscript.

**R: Thank you.**

Reviewer #1 (Remarks to the Author):

I acknowledge all the efforts undertaken by the authors efforts to provide the missing experiments and data suggested. This represents a significant work which is appreciated. The authors also answered professionally and with details to the comments.

Some divergence between authors and the reviewer remain about point 1c (ii) but this does not justify another round of revision since other experiments seem to converge to the same conclusion. I thus accept as is.

Only one last request: the student t-test is applied for data plotted in fig 3c and 5e but this test is not appropriate for these data. The assumptions for t-test are: a normal distribution and same variance between groups . This is not the case here with so little datapoints. Best would be a Kruskall Wallis followed by post-hoc Dunn's test (see for instance: https://www.statskingdom.com/kruskal-wallis-calculator.html). For Graph 3c, it means for the experiment with ORC1b-GFP: control 4h-Bort4h: P=0.09 and control 6h-MLN 6h: P=0.0017. Note that the test should be done on the raw values and not the normalised values (where control=1). Please correct and comment accordingly the result in the main text. Please do the same for data fig 5e and modify the description in the legend.

I congratulate the authors for the very nice work and all the efforts invested since the first submission.

**REVIEWERS' COMMENTS**

Reviewer #1 (Remarks to the Author):

I acknowledge all the efforts undertaken by the authors efforts to provide the missing experiments and data suggested. This represents a significant work which is appreciated. The authors also answered professionally and with details to the comments.
Some divergence between authors and the reviewer remain about point 1c (ii) but this does not justify another round of revision since other experiments seem to converge to the same conclusion. I thus accept as is.

Only one last request: the student t-test is applied for data plotted in fig 3c and 5e but this test is not appropriate for these data. The assumptions for t-test are: a normal distribution and same variance between groups. This is not the case here with so little datapoints. Best would be a Kruskall Wallis followed by post-hoc Dunn's test (see for instance: https://www.statskingdom.com/kruskal-wallis-calculator.html). For Graph 3c, it means for the experiment with ORC1b-GFP: control 4h-Bort4h: P=0.09 and control 6h-MLN 6h: P=0.0017. Note that the test should be done on the raw values and not the normalised values (where control=1). Please correct and comment accordingly the result in the main text. Please do the same for data fig 5e and modify the description in the legend.

**R: Following your indication we have applied the Kruskal-Wallis test with Dunn's multiple comparisons for the raw data of Fig. 3c and Fig. 5e. This is indicated in the legends and the details provided in the Supplementary Data 1 tables.**

I congratulate the authors for the very nice work and all the efforts invested since the first submission.

**R: Thank you.**
